# HDAC3 Inhibition Stimulates Myelination in a CMT1A Mouse Model

**DOI:** 10.1007/s12035-022-02782-x

**Published:** 2022-03-23

**Authors:** Robert Prior, Stijn Verschoren, Katlijn Vints, Tom Jaspers, Elisabeth Rossaert, Yvonne E. Klingl, Alessio Silva, Nicole Hersmus, Philip Van Damme, Ludo Van Den Bosch

**Affiliations:** 1 Department of Neurosciences, Experimental Neurology and Leuven Brain Institute (LBI), KU Leuven - University of Leuven, B-3000 Leuven, Belgium; 2 Laboratory of Neurobiology, VIB, Center for Brain & Disease Research, Campus Gasthuisberg O&N5, Herestraat 49, box 602, B-3000 Leuven, Belgium; 3 Electron Microscopy Platform & VIB BioImaging Core, Herestraat 49, B-3000 Leuven, Belgium; 4 Neurology, University Hospitals Leuven, B-3000 Leuven, Belgium

**Keywords:** HDAC3 inhibition, CMT1A, Schwann cell, Myelination, Inflammation, RGFP966

## Abstract

Charcot–Marie–Tooth disease (CMT) is the most common inherited peripheral neuropathy, with currently no effective treatment or cure. CMT1A is caused by a duplication of the *PMP22* gene, which leads to Schwann cell differentiation defects and dysmyelination of the peripheral nerves. The epigenetic regulator histone deacetylase 3 (HDAC3) has been shown to negatively regulate myelination as well as its associated signaling pathways, PI3K-AKT and MAPK-ERK. We showed that these signaling pathways are indeed downregulated in the C3-PMP22 mouse model, similar to what has been shown in the CMT1A rat model. We confirmed that early postnatal defects are present in the peripheral nerves of the C3-PMP22 mouse model, which led to a progressive reduction in axon caliber size and myelination. The aim of this study was to investigate whether pharmacological HDAC3 inhibition could be a valuable therapeutic approach for this CMT1A mouse model. We demonstrated that early treatment of CMT1A mice with the selective HDAC3 inhibitor RGFP966 increased myelination and myelin g-ratios, which was associated with improved electrophysiological recordings. However, a high dose of RGFP966 caused a decline in rotarod performance and a decline in overall grip strength. Additionally, macrophage presence in peripheral nerves was increased in RGFP966 treated CMT1A mice. We conclude that HDAC3 does not only play a role in regulating myelination but is also important in the neuroimmune modulation. Overall, our results indicate that correct dosing of HDAC3 inhibitors is of crucial importance if translated to a clinical setting for demyelinating forms of CMT or other neurological disorders.

**Supplementary Information:**

The online version contains supplementary material available at 10.1007/s12035-022-02782-x.

## Introduction

The major form of Charcot–Marie–Tooth disease is type 1A (CMT1A), which is caused by a duplication of a segment on chromosome 17p11.2 containing the gene encoding *peripheral myelin protein 22* (*PMP22*) [1–3]. *PMP22* is essential for correct Schwann cell myelin development, membrane architecture formation, and maintenance [4, 5]. CMT1A patients have a reduction in large myelinated axon fibers with prominent dysmyelination of the largest peripheral nerves [6]. Clinically, this results in mild to severe muscular atrophy and sensory abnormalities in the distal regions of the body [6]. Studies using the CMT1A rat model suggest that defects in lipid metabolism and myelination arise during early postnatal development [7]. Moreover, a disturbed balance of the phosphatidylinositol 3‑kinase (PI3K)/protein kinase B (AKT) and the mitogen activated protein kinase (MAPK)-extracellular-signal-regulated kinase 1/2 (ERK1/2) signaling pathways (also known as the Ras-Raf-MEK-ERK1/2 pathway) seem to lead to Schwann cell differentiation and myelination deficits in young CMT1A rats [8].

The initial steps of myelination are triggered through a number of biological cues, such as the interaction with the transmembrane axonal growth factor neuregulin 1, type III (NRG1, type III) which binds to the cell membrane receptor tyrosine protein kinase erbB-2 and erbB-3 (ERBB2–ERBB3), as well as signaling through integrin–laminin, and GPR126 receptor binding (reviewed in [9]). Together, these pathways lead to PI3K–AKT and MAPK–ERK1/2 activation, which is critical for myelination. These signaling cascades activate a series of transcriptional promyelinating regulators, which ultimately stimulate the transcription of genes regulating myelin biogenesis [10]. PI3K–AKT signaling is key during differentiation of Schwann cells to a myelinating phenotype [11], while the MAPK–ERK1/2 signaling pathway seems to be essential for active myelination during development and regeneration after acute nerve injury [12, 13]. In addition to these signaling pathways, the initiation of myelinogenesis depends on a number of intrinsic and extrinsic factors, such as upregulation of cyclic adenosine monophosphate (cAMP) and axo-glial communication, respectively. The latter also determines myelin thickness, which is essential for saltatory nerve conduction in nerve fibers [14].

In the peripheral nervous system (PNS), the myelination process is controlled by epigenetic modifiers known as histone deacetylase enzymes (HDACs) [15, 16]. The major myelination transcription factor, early growth response 2 (EGR2/KROX20), is transcriptionally repressed by one of the main chromatin remodeling complexes in cells, the nucleosome remodeling and deacetylase (NuRD) complex [15, 16]. The NuRD complex is an ATP-dependent chromatin remodeling complex, composed of a number of core components, including HDAC1 and HDAC2 [17]. In addition, other class I and IIa HDACs (HDAC3, 4, and 5) are key regulators of Schwann cell myelination [14, 18]. HDAC3 seems to be a negative regulator of EGR2, as HDAC3 knockdown resulted in an upregulation of EGR2 protein and mRNA expression, thereby dramatically upregulating myelination [18]. Moreover, HDAC3 has also been shown to repress activation of the ERK1/2 signaling pathway [18, 19].

The aim of this study was to determine whether inhibition of HDAC3 enzymatic function using the HDAC3 inhibitor (HDAC3i) RGFP966 could be a therapeutic approach in a CMT1A mouse model. We observed that the HDAC3i indeed induced myelination through the activation of the PI3K–AKT and MAPK–ERK1/2 signaling pathways, leading to a significant increase in sciatic nerve myelin proteins’ expression, nerve conduction velocities, and compound muscle action potentials (CMAPs) in the CMT1A mice. However, a dichotomous dose-dependent muscle-specific phenotype was observed in both wild-type and CMT1A treated mice. In addition, an increase in the presence of endoneural macrophages was noted in the peripheral nerves of high-dose treated CMT1A mice. Altogether, our data demonstrate that HDAC3 plays a multifaceted role in myelination, in muscle physiology, and in the neuroimmune response.

## Materials and Methods

### Animals

All procedures were conducted in accordance with the ethical standards for experiments on animals established and approved by the Animal Ethics Committee of the KU Leuven. Experiments were conducted under the ethical committee approval n° 104/2017. Mice were kept in filter top cages in controlled environment with a room temperature between 20 and 21 °C, humidity between 50 and 60%, and a light–dark cycle of 12 h.

The C3-PMP22 (Tg(PMP22)C3) mouse model [20] which was obtained from the Academisch Medisch Centrum (University of Amsterdam, the Netherlands) was maintained in a C57BL6/J genetic background. Genotyping was conducted using digital droplet PCR (ddPCR) (see below) until heterozygous animals were identified, and then these mice were crossbred with wild-type (Wt) mice, resulting in either heterozygous or Wt offspring. The genotyping from these litters was done by standard PCR to distinguish between C3 mice and littermate Wt mice. For PCR analysis, genomic DNA was purified from a small section of mouse ear or tail by lysing the tissue overnight at 55 °C with proteinase K (20 mg/ml; Roche, Basel, Switzerland) in a lysis buffer composed of 0.2% SDS, 200 mM NaCl, 100 mM Tris–HCl (pH 8.5), and 5 mM EDTA. Samples were then centrifuged, and DNA was precipitated using isopropanol and pelleted by centrifugation. Pellets were subsequently washed in 75% ethanol and dissolved in Tris–EDTA buffer containing 0.1 mM EDTA and 10 mM Tris–HCl (pH 7.5). Primers used for genotyping were purchased from IDT solutions (Leuven, Belgium) and were the following:

- PMP22 FWD primer: 5′- TGG TGA TGA GAA ACA GT -3′.
- PMP22 REV primer: 5′- TGA TTC TCT CTA GCA ATG GA -3′.
- IL FWD primer: 5′- CTA GGC CAC AGA ATT GAA AGA TCT -3′.
- IL REV primer: 5′- GTA GGT GGA AAT TCT AGC ATC C -3′.

PCR program cycle conditions were as follows: (1) 3 min at 94 °C, (2) 10 s at 94 °C, (3) 10 s at 53.8 °C, (4) 10 s at 72 °C, and (5) 5 min at 72 °C, and then the run was held at 15 °C until the plate was removed from the PCR thermal cycler. Steps 2–4 were set to a cycle of 30 times. The subsequent PCR product was ran on a 2% agarose gel, stained using SYBR Safe DNA Gel Stain (Thermo Fisher Scientific, Waltham, MA, USA) and visualized using a transilluminator (UVP Visi-Blue™ Transilluminator, Thermo Fisher Scientific).

### Digital Droplet PCR

The concentration of genomic DNA was determined using NanoDrop (Thermo Fisher Scientific). Subsequently, a restriction enzyme digest was made using FastDigest Buffer (10 × ; Thermo Fisher Scientific), FastDigest HAEIII (10 U; Thermo Fisher Scientific), 500 ng genomic DNA, and nuclease-free water in a total of 50 μl and incubated at 30 °C overnight or 37 °C for 60 min. PCR reaction mix was formulated by combining ddPCR supermix for probes (no UTP) 2 × (Bio-Rad, Hercules, USA), the 20 × Taqman assay, 2 ng of diluted cDNA, and nuclease-free water. Samples were then mixed by vortexing in short pulses and centrifuged briefly. Assembled reaction mixtures were loaded into the DG8™ Cartridge (Bio-Rad, #1,864,008) followed by 70 μl of Droplet Generation Oil for Probes (Bio-Rad, #1,863,005). The QX200™ Droplet Generator (Bio-Rad) was used for droplet generation according to the manufacturer’s guidelines and then loaded to ddPCR plates (Bio-Rad) and heat sealed with foil. The PCR digest was run on PCR thermal cycler with the following program: (1) 10 min at 95 °C, (2) 30 s at 95 °C, (3) 1 min at 58 °C, and (4) 10 min at 98 °C, (5) and then the run was held at 12 °C until the plate was removed from the PCR thermal cycler. Steps 2–4 were set to a cycle of 39 times. The subsequent loaded ddPCR plate was read using QX200 Droplet Reader and QuantaSoft Software (Bio-Rad).

The human-specific ddPCR copy number variation assay for *PMP22* (dHsaCP2500327; Bio-Rad, #10031240) was used to determine the copy number of human *PMP22* in the CMT1A mice. The reference gene used in combination with the human *PMP22* assay was the commercially established *AP3B1* (dHsaCP1000001; Bio-Rad, #10031245) antibody ddPCR copy number variation assay.

### HDAC3 Inhibitor Treatment

For treatment, the selective HDAC3 inhibitor (HDAC3i) RGFP966 (99.5% purity; Asclepia, Destelbergen, Belgium) was used as reported before [18, 21]. RGFP966 has a > 200-fold selectivity towards HDAC3 versus other HDACs; HDAC3 IC_50_ at 0.08 μM versus a HDAC2 IC_50_ at 17.7 μM and a HDAC1 IC_50_ at 28.7 μM [21] (see Additional File 1, Figure S1). Stock solutions of 5 mg/ml or 10 mg/ml RGFP966 were prepared in DMSO. These solutions were diluted 1/10 in sterile injectable water (Aqua ad iniectabilia, Mini-Plasco connect®) containing 30% hydroxyl-beta-cyclodextrin and 0.1 M acetate (pH 5.4) until a 10% DMSO concentration was reached. RGFP966 (either 5 mg/kg or 10 mg/kg body weight) was administered starting at P6. For control mice, 10% DMSO was used in the subsequent steps. The first 7 days, the compound was administered subcutaneously once daily, after which the administration route was switched to intraperitoneal (I.P.) injections, every other day for 2 weeks (7 injections in total). No side effects were observed in the control treated animals. No inclusion or exclusion criteria were applied to the mice. Males and females were included in the study. As genotype identification was not always possible before postnatal day 6, the treatment of genotypes was blinded at certain times, leading to slight disparities in the sample sizes. During phenotyping, investigators were blinded to genotype and treatment.

### Motor Phenotyping

Motor phenotyping was conducted 2 days after the treatment was completed, starting from postnatal day 30. Muscle force of the mice was determined using a grip strength meter (Columbus Instruments, Columbus, USA). Overall grip strength was assessed using a metal mesh assembly to assess the combined grip strength of the fore- and hindlimbs. Mice were held by their tail and gently pulled backwards while gripping the metal mesh, until they lost their grip. The average of 5 trials per animal was determined. Motor performance was assessed using a Rotarod (Ugo Basile, Gemonio, Italy), with a constant speed of 15 rpm. The latency to fall was recorded to a maximum of 300 s. A rest period of 5 min was given between each trial. A total of 3 trials was recorded, and the mean of these trials is used in graphs.

### Electrophysiology

Initial electrophysiology follow-up characterization of the C3 phenotype was conducted at 2–6 months of age. An additional CMAP tracing is qualitatively shown as part of the initial follow-up at postnatal day 35 to illustrate earlier electrophysiology abnormalities in the C3 versus Wt littermate mice. For the HDAC3i treatment study, electrophysiology was conducted after motor phenotyping was completed, between postnatal days 35 and 36. For CMAP recordings, mice were anaesthetized using 3% isoflurane under a 2.5 l/min oxygen flow. Once sedated, they were kept under constant flow of 2-3% isoflurane and placed on a 37 ± 0.5 °C heating pad to maintain body temperature. Recordings were performed using 0.4 mm platinum coated sub-dermal needle electrodes (Technomed Europe, Maastricht, Netherlands) and the Natus ultraPro S100 (Natus Medical Incorporated, Pleasanton, USA) as described before [20, 22, 23]. The sciatic nerve was stimulated by placing the anode and cathode proximal of the sciatic notch, 0.5 cm apart from each other. The reference electrode was placed at the ankle of the same leg, and the measuring electrode was positioned parallel with the gastrocnemius muscle, subdermally. The distance between these two electrodes was measured to calculate nerve conduction velocities (NCVs). The ground electrode was positioned at the base of the tail. The stimulating current was increased gradually until a stable supramaximal response was generated. The highest supramaximal responses were taken for each mouse out of 3 recordings for each time-point and used in the graphs. For latency and NCV calculations, a mean of the 3 recordings per mouse was used in the graphs.

### Histopathology

Histology was performed at the end of phenotyping (postnatal day 37), when mice were sacrificed. First, mice were anaesthetized by an I.P. injection with sodium pentobarbital (200 mg/kg: Dolethal, Vetoquinol). When the mice were non-responsive to pressure applied to their foot or tail, they were transcardially perfused with PBS. For electron microscopy, mice were additionally perfused with 2.4% glutaraldehyde, 0.1 M Na-cacodylate and 4% paraformaldehyde dissolved in Milli-Q® ultrapure water (Merck, Darmstadt, Germany). Fixation solution was filtered through a 0.2-μm vacuum filter bottle system (Corning®, Lasne, Belgium, #430,771) before use. Isolated sciatic nerves were further post-fixed in Milli-Q® water containing 2.4% glutaraldehyde, 0.1 M Na-cacodylate, and 4% paraformaldehyde overnight at 4 °C. The following day, samples were washed twice with a 0.1 M Na-cacodylate buffer and washed for 30 min. Samples were then transferred to glass vials and post-fixed in 2% osmium tetroxide for 120 min. Samples were subsequently dehydrated as follows: 50% EtOH 15 min (× 2), 70% EtOH 15 min (× 2), 96% EtOH 15 min (× 2), and 100% EtOH 15 min (× 2).

Impregnation was as follows: 20 min propylene oxide (× 2), 60 min at room temperature 1:1 propylene oxide/agar, and then 1:2 propylene oxide/agar overnight at room temperature. Samples were then placed in fresh agar for 6–8 h at room temperature, after which they were placed in a rubber mold containing fresh agar. Samples were oriented so that the most distal region of the sciatic nerve was facing the edge of the molds. To harden the samples, they were placed in a 60 °C oven for 48 h. Samples were trimmed using a Leica EM TRIM (Leica Microsystems), and semi-thin (0.5 μm) transverse sections were cut using a Leica ultra-microtome (Leica Ultracut S, Leica Microsystems). Finally, the sections were stained with 1% toluidine blue at 80 °C for 1 min.

Ultra-thin sections (70 nm) were cut and collected on 75-mesh grids (Van Loenen Instruments, Zaandam, Netherlands) followed by post-staining with 1% uranyl acetate solution and lead citrate before being imaged with a JEOL JEM-1400 Transmission Electron Microscope (Joel Ltd., Akishima, Tokyo, Japan) operated at 80 kV. For statistical analyses, nerve fibers from up to 4 animals per group were used. Each sciatic nerve section was analyzed at × 800 magnification. The ImageJ plugin “GRatio” was used to analyze *g*-ratios at randomly selected fibers. The investigator was blinded from the genotype and treatment during the analysis. At least 95 fibers were randomly selected per animal at the central region of the sciatic nerve for all animals. For pathological observations, ranges up to × 5000 magnification were used.

For immunohistochemistry of the brachial plexus nerve (specifically, the medial anterior thoracic nerve), animals were transcardially perfused with PBS. Isolated brachial plexus nerves were snap-frozen in isopentane that had been cooled by immersion in liquid nitrogen and placed on dry ice. Samples were then stored at − 80°C until further use. The nerves were later embedded in optimal cutting temperature compound (OCT compound, Fisher Scientific) and stored at − 80 °C until sectioning. Transverse sections of the nerve were cut at a thickness of 20 μm using a cryostat (Cryostar NX70, ThermoFisher Scientific). The average of at least 3 slices was analyzed per animal at an 80 μm depth difference apart from each slice area in each brachial plexus section to avoid analysis of overlapping slices. Sections were collected on a coverslip glass and air-dried for 1–2 h. Sections were then post-fixed in 4% PFA for 15 min at room temperature, followed by immersion in 100% methanol for 6 min at − 20 °C. After fixation, samples were washed twice with PBS and once with PBST (0.3% Triton X-100 (Sigma, St. Louis, USA)), each for 5 min. Samples were then blocked in 5% normal donkey serum (Sigma) and 5% goat serum (Sigma) in PBS for at least 1 h. Sections were then incubated with primary antibodies overnight at 4 °C. Primary antibodies F4/80 (Bio-Rad, #MCA497G) and CD34 (ThermoFisher Scientific, #14–0341-85) were used at a 1:300 and 1:1000 dilution, respectively. After incubation, slides were washed three times for 5 min with PBS, and appropriate secondary antibodies were used at a 1:1000 dilution in blocking buffer for 1 h. Following this, slides were washed three times with PBS for 5 min, after which they were mounted with ProLong® Gold antifade reagent containing DAPI (Invitrogen, Life Technologies, Carlsbad, USA). The amount of F4/80+ cells per brachial plexus nerve section was quantified as the level of macrophages per nerve section, and each time a F4/80+ cell touched a CD34+ cell, this was taken as an interaction.

For immunohistochemistry of gastrocnemius muscle, transverse sections of the muscle were cut at a thickness of 20 μm using a cryostat, allowed to dry for 1–2 h and then post-fixed for 20 min with 4% PFA at room temperature. Sections were then blocked for 1 h and incubated with wheat germ agglutinin primary antibody conjugated to Alexa Fluor-488 (Invitrogen, #W11261). Slides were washed three times for 5 min with PBS before mounting with ProLong® Gold antifade reagent without DAPI (Invitrogen, #P36934). All images were acquired using the Leica SP8 X confocal microscope and analyzed using ImageJ.

### Western Blot Analysis

For analysis of whole-protein lysates, snap-frozen whole sciatic nerve tissues were lysed in ice-cold RIPA buffer (50 mM Tris–HCl (pH 7.5), 0.5% SDS, 150 mM NaCl, 0.5% Na-deoxycholic acid, and 1% NP-40), supplemented with complete EDTA-free protease inhibitor cocktail (Roche, Basel, Switzerland). Tissues were homogenized using lysing matrix D beads (MP Biomedical, Illkirch Cedex, France) and a tissue homogenizer instrument (FastPrep24-5G- MP Biomedical, USA). For nuclear and cytoplasmic fractionation of sciatic nerve tissue, samples were prepared using the NE-PER™ Nuclear and Cytoplasmic Extraction kit (Thermo Fisher Scientific, #78,835) according to the manufacturer’s guidelines. Protein concentration measurements and Western blot preparations were performed as described before [22, 24]. The following primary antibodies were diluted in 5% BSA blocking buffer: myelin basic protein (Millipore, #MAB386) 1:3000, phosphorylated-AKT (Cell Signaling Technology, #9271S) 1:1000, AKT (Cell Signaling Technology, #9272) 1:1000, phosphorylated-ERK1/2 (Cell Signaling Technology, #4370) 1:1000, ERK1/2 (Cell Signaling Technology, #4695) 1/1000, histone H4 (Abcam, #ab10158) 1/1000, CSF-1-R (Abcam, #ab271294) 1/500, and PMP22 (Origene, #TA808964) 1:1000, or acetylated histone H3 (Cell Signaling Technology, #4353S) 1:500. Secondary antibodies conjugated with horseradish peroxidase (Agilent Technologies (Dako); 1:5000, 1 h, RT) were used prior to detection with enhanced chemiluminescence substrate (ECL substrate; ThermoFisher Scientific) and a LAS 4000 Image Analyzer (GE Healthcare, Little Chalfont, UK). Total protein levels were visualized using No-Stain™ Protein Labeling Reagent (ThermoFisher Scientific, #A44717). Luminescent signals were analyzed with ImageQuant TL software (GE Healthcare). The band intensities from the proteins of interest were normalized to the total amount of protein loaded in each lane. For representation in figures, a lane of an equal molecular weight is used.

### Statistics

A priori power analyses were conducted using the software G*Power version 3.1.7. Adequate power (1–β-error) was defined as ≥ 80% with an alpha error of 5%. All other statistical analyses were performed using GraphPad Prism software version 9 (GraphPad software Inc., California, USA) or MS Excel. All data were first checked for normality in GraphPad Prism to select the appropriate statistical test. Additionally, where appropriate, data were checked for outliers using GraphPad Grubbs analysis. The unpaired two-tailed Student’s *t* test was used for the comparison of two means; one-way ANOVA tests were performed for the multiple group analysis. Data from in vivo studies and Western blot are presented as means ± SD, and data from and histopathological analyses are presented as means ± SEM. Statistical significance was set at the following: *p < 0.05, **p < 0.01, ***p < 0.001, and ****p < 0.0001.

## Results

### CMT1A Mice Display Severe Myelin Defects

**Fig. 1:**
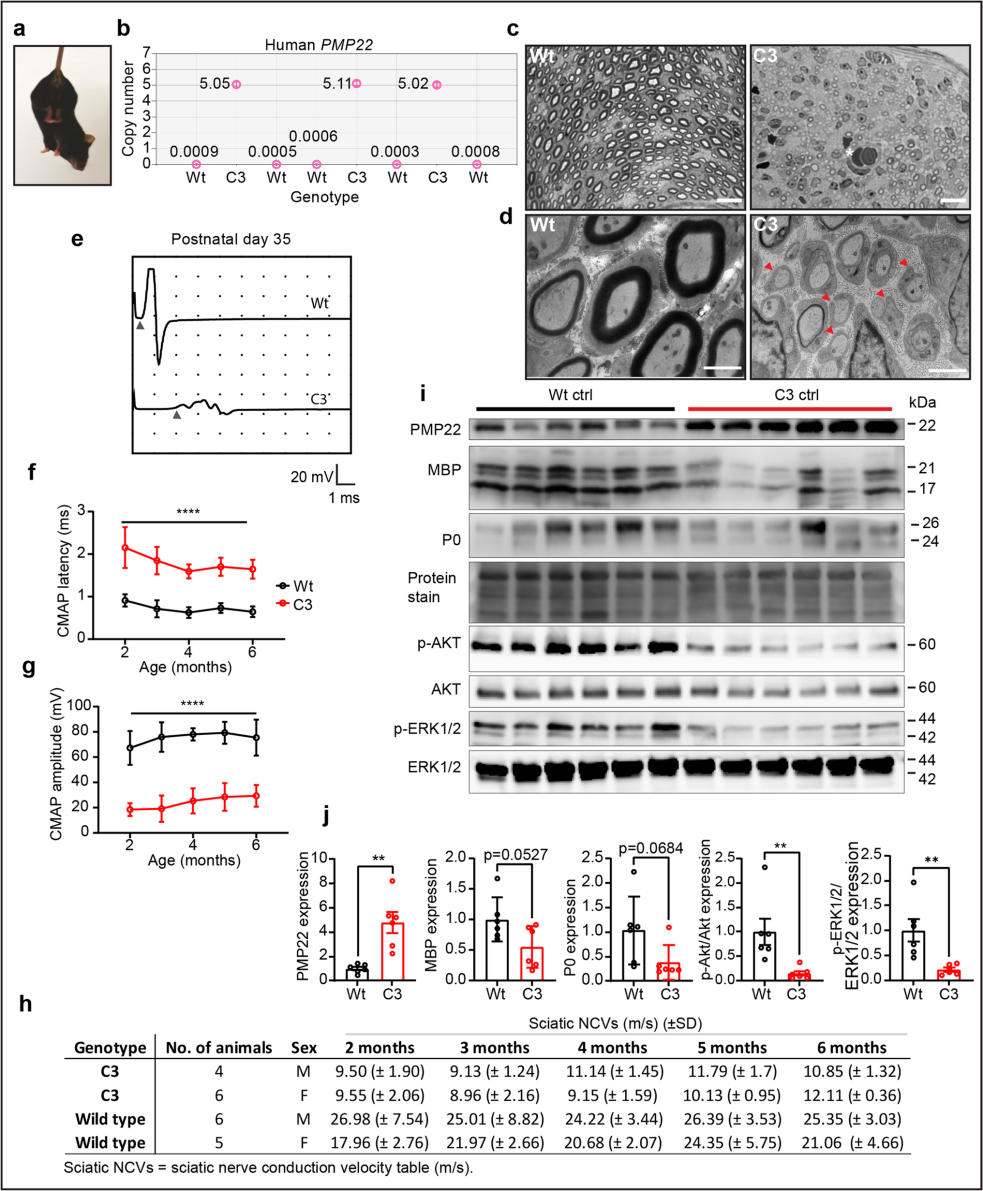
C3 mice have 5 copies of human *PMP22*, show postnatal myelination defects, and have electrophysiological abnormalities throughout development. **a** Adult C3 mice show a limb clasping behavior when held in suspension by their tail. The C3 mouse on the image was 1 year old. **b** ddPCR on genomic DNA isolated from Wt and C3 mice. 0 positive copy numbers of human *PMP22* were detected in Wt mice (*n* = 5 mice) in comparison to the 5 detected copies of human *PMP22* in the C3 mice (*n* = 3 mice). **c** Toluidine blue-stained semi-thin sections of sciatic nerve isolated from postnatal day 6 pups. Wt pups have initiated myelination normally by postnatal day 6. In comparison, C3 mice have Schwann cell developmental myelin abnormalities (indicated by white asterisk). Scale bar,: 10 μm. **d** Electron microscopy images of the pups used in **c**. On the left, Wt pups have initiated myelination. In the C3 pup (right), a large delay in initiating myelination is visible in the C3 pup electron micrograph by Schwann cells just starting to associate with axons. There are approximately twice as many Schwann cells in the C3 image in comparison to the Wt condition, which possibly indicates Schwann cell hyperplasia. Red arrow heads indicate Schwann cells just initiating myelination. Scale bar: 2 μm. **e** Representative electrophysiological traces of sciatic nerve CMAP motor recordings at postnatal day 35 which are severely reduced in C3 mice compared to Wt mice. Arrowheads indicate where the action potential starts, which was used for determining the latency. **f** Prolonged CMAP latencies and **g** a reduction in CMAP amplitude recordings in C3 mice in comparison to Wt as a function of age. **h** Summary of nerve conduction velocity data from the mice used in the follow-up. **i** Protein expression of PMP22, myelin basic protein (MBP), myelin protein zero (P0), and PI3K–AKT and MAPK/ERK1/2 pathways. **j** Quantification of Western blots in **i**. A two-way ANOVA test with Šidák multiple comparisons tests was used in panels **e** and **f** to compare Wt (*n* = 11 mice) and C3 mice (*n* = 10 mice). Data are represented as the mean ± SD (**** *p* < 0.0001). In **i** and **j**, 6-month-old Wt (*n* = 6 mice) and C3 mice (*n* = 6 mice) were used for analysis. A Students *t* tests was used for statistical analysis of Wt and C3 groups in **j**. Data are represented as the mean ± SEM (** *p* < 0.01)

Dysregulated myelination accompanied by a reduction in axon caliber size are core histopathological features of CMT1A patient nerve biopsies [25]. In the rat model of CMT1A, Schwann cell developmental defects have been described before [8, 26]. Therefore, we investigated the process of early postnatal myelinogenesis in the C3-PMP22 CMT1A mice [27], referred to as C3 mice throughout this study. These mice display strong CMT-like symptoms, such as a clear limb clasping behavior phenotype at 1 year of age (Fig. 1a). Using ddPCR, we determined that they have 5 copies of human *PMP22* integrated in their genome (Fig. 1b), which is more than the 3–4 copies initially reported [27].

To investigate potential early myelination defects in these mice, we assessed postnatal myelin morphology and performed electrophysiological recordings in the sciatic nerve up to 6 months of age. At postnatal day 6, we observed that wild-type littermate control pups (Wt) had initiated myelination normally, while the C3 mice displayed clear abnormal Schwann cell myelination (Fig. 1c). A delay in initiating myelination and Schwann cell hyperplasia was observed in the C3 mice at an electron microscopic level (Fig. 1d). It is known that CMT1A patients have an increased number of non-myelinating Schwann cells in their peripheral nerves [16, 28].

In vivo nerve conduction recordings at postnatal day 35 showed prolonged compound CMAP latencies and reduced amplitudes (Fig. 1e). A follow-up of the C3 mice showed that these electrophysiological abnormalities persisted as far as 6 months of age in comparison to their Wt littermates (Fig. 1f, g). Nerve conduction velocities (NCVs) were strongly downregulated in comparison to the Wt mice throughout development (Fig. 1h). CMAP latencies and NCVs relate to the degree of myelination in the nerve fibers and/or the distance between the nodes of Ranvier, whereas CMAP amplitudes give an indication of the number of sciatic nerve axons propagating action potentials to the connecting peripheral muscle. These neurological deficits were most severe in young mice but slightly improved with age (Fig. 1f, g, h), in line with what was originally described [27]. Additionally, in 6-month-old mice, PMP22 protein levels were shown to be highly upregulated, while the expression of the major myelin proteins myelin basic protein (MBP) and myelin protein zero (P0) levels was shown to be approximately 50% reduced in the C3 mice (Fig. 1i, j). Furthermore, the activation of PI3K–AKT and MAPK/ERK1/2 signaling activity (measured by phosphorylated-AKT (p-AKT) and phosphorylated-ERK1/2 (p-ERK1/2))—which are essential for myelination—were shown to be significantly downregulated in the C3 mouse model (Fig. 1i, j). Overall, our results demonstrate that the C3 mice have altered myelin protein expression in adult mice and severe developmental myelination defects.

### Pharmacological Inhibition of HDAC3 Increases Myelin Synthesis and Signaling Pathways Regulating Myelination in Wt Mice

HDAC3 has been shown to be a master regulator in peripheral nerve myelin growth and regeneration [18]. Additionally, Rosenberg et al. demonstrated that HDAC3 regulates the transition from a myelinating Schwann cell to a homeostatic myelinating Schwann cell [29]. To stimulate myelination during development, our aim was to reduce HDAC3 catalytic enzymatic activity. Therefore, we pharmacologically inhibited HDAC3 using the benzamide-based, selective HDAC3 inhibitor (HDAC3i) RGFP966, which has a > 200-fold selectivity towards HDAC3 versus other HDACs [21].

**Fig. 2:**
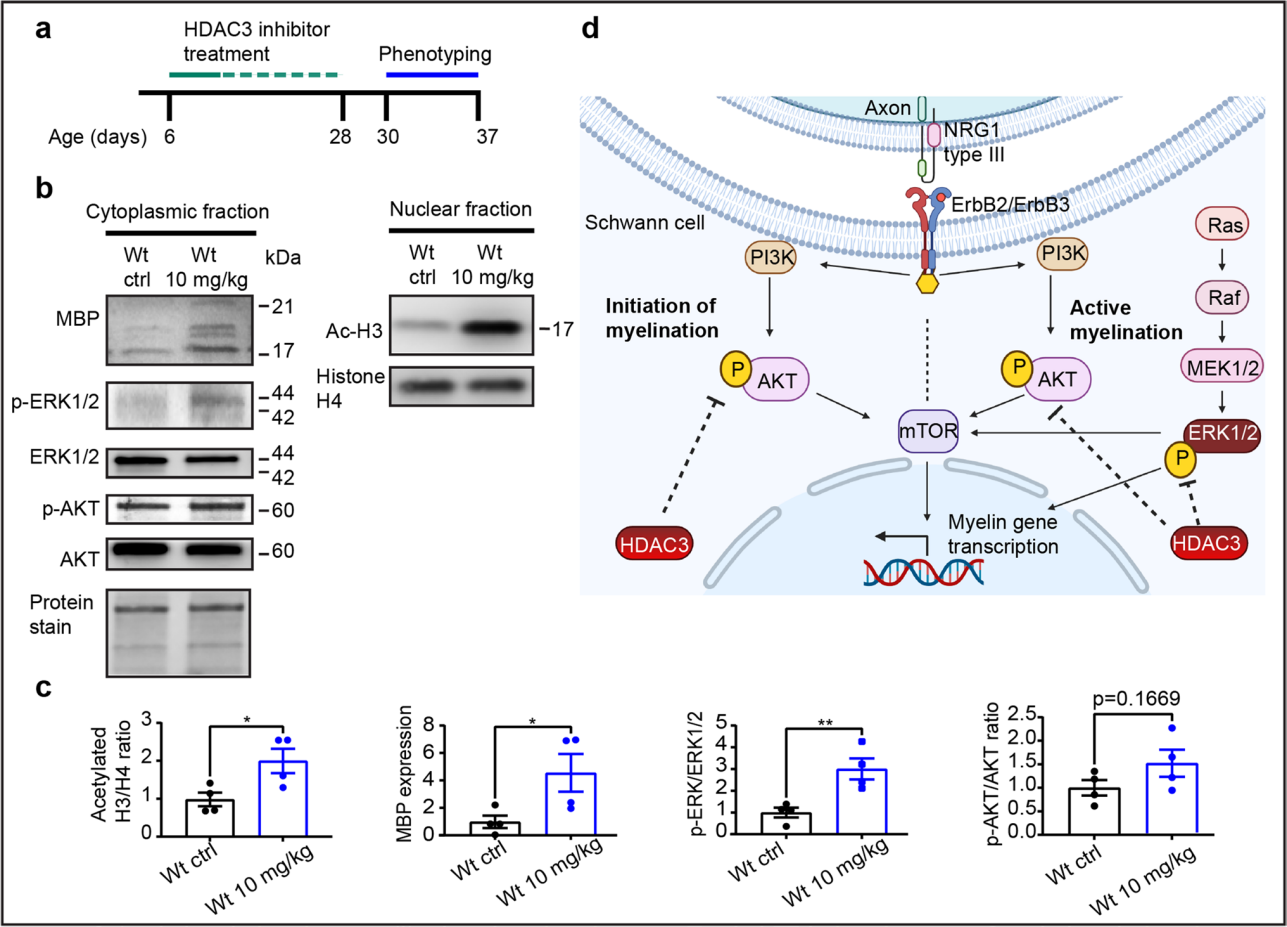
Inhibiting HDAC3 enzyme activity increases AKT-ERK1/2 signaling and myelin basic protein expression. **a** Schematic overview of the treatment scheme for the C3 and wild-type mice (Wt) with RGFP966. Treatment consisted of 1 week of daily subcutaneous injections, followed by I.P. injections given every second day for a total of 2 weeks (7 I.P. injections in total). Treatment is represented by a full green line followed by 7 green dashes representing the periodic distribution of treatment injections. Phenotyping is represented as a solid blue line, representing a week-long examination of the animals’ motor phenotype starting from postnatal day 30, 2 days after the treatment has been completed. This was followed by electrophysiological examinations and, lastly, by sacrificing of the animals for histopathological examinations. **b** Western blot of sciatic nerve cytoplasmic and nuclear-enriched fractions from a Wt control (Wt ctrl) and a Wt treated mouse with 10 mg/kg RGFP966. Acetylated histone H3 (Ac-H3) and histone H4 are shown in the nuclear-enriched fraction. Histone H4 was used as loading control for the nuclear fraction and for quantifications. MBP, p-ERK1/2, ERK1/2, p-AKT, and AKT are shown in the cytoplasmic-enriched fractions. A total protein stain was used as loading control. **c** Quantification of Western blots shown in **b** (*n* = 4 mice for each group). All quantifications are expressed as fold change compared to Wt ctrl. **d** Schematic overview showing PI3K/AKT and MAPK/ERK1/2 signaling pathways during the initiation and active myelination, based on [12] and reviewed in [9]. Image was created with http://www.BioRender.com. HDAC3 dotted lines are based on protein expression shown in **b-c**, as well as from He et al. [18]

To validate whether RGFP966 can stimulate myelination, Wt pups were treated with RGFP966 for 3 weeks starting from postnatal day 6, which was followed by phenotyping starting at postnatal day 30 and subsequent histopathological analysis at postnatal day 37 (overview of treatment schedule illustrated in Fig. 2a). Sciatic nerves were collected at the end of phenotyping, and tissue protein lysates were separated into cytoplasmic and nuclear-enriched fractions for Western blot analysis.

Acetylated histone H3, the major target of the HDAC3 enzyme, was significantly upregulated in nuclear fractions (Fig. 2b, c), confirming reduction of HDAC3 catalytic activity in vivo. To assess myelination, we analyzed the levels of MBP and the signaling activity p-AKT and p-ERK1/2. Interestingly, we observed an upregulation of MBP protein isoforms 17–21 kDa in the cytoplasmic-enriched fractions in HDAC3i treated mice (Fig. 2b, c). Moreover, we observed a significant increase in p-ERK1/2 levels in cytoplasmic fractions of Wt 10 mg/kg HDAC3i treated sciatic nerves but did not record a significant change the levels of p-AKT (Fig. 2b, c). Therefore, our results show an activation of pro-myelination signaling by the HDAC3i treatment, indicating that RGFP966 stimulates myelin protein synthesis in vivo in postnatal Wt pups (overview illustrated in Fig. 2d).

### Pharmacological Inhibition of HDAC3 Increases Myelin Protein Synthesis and Signaling Pathways in C3 Mice

**Fig. 3:**
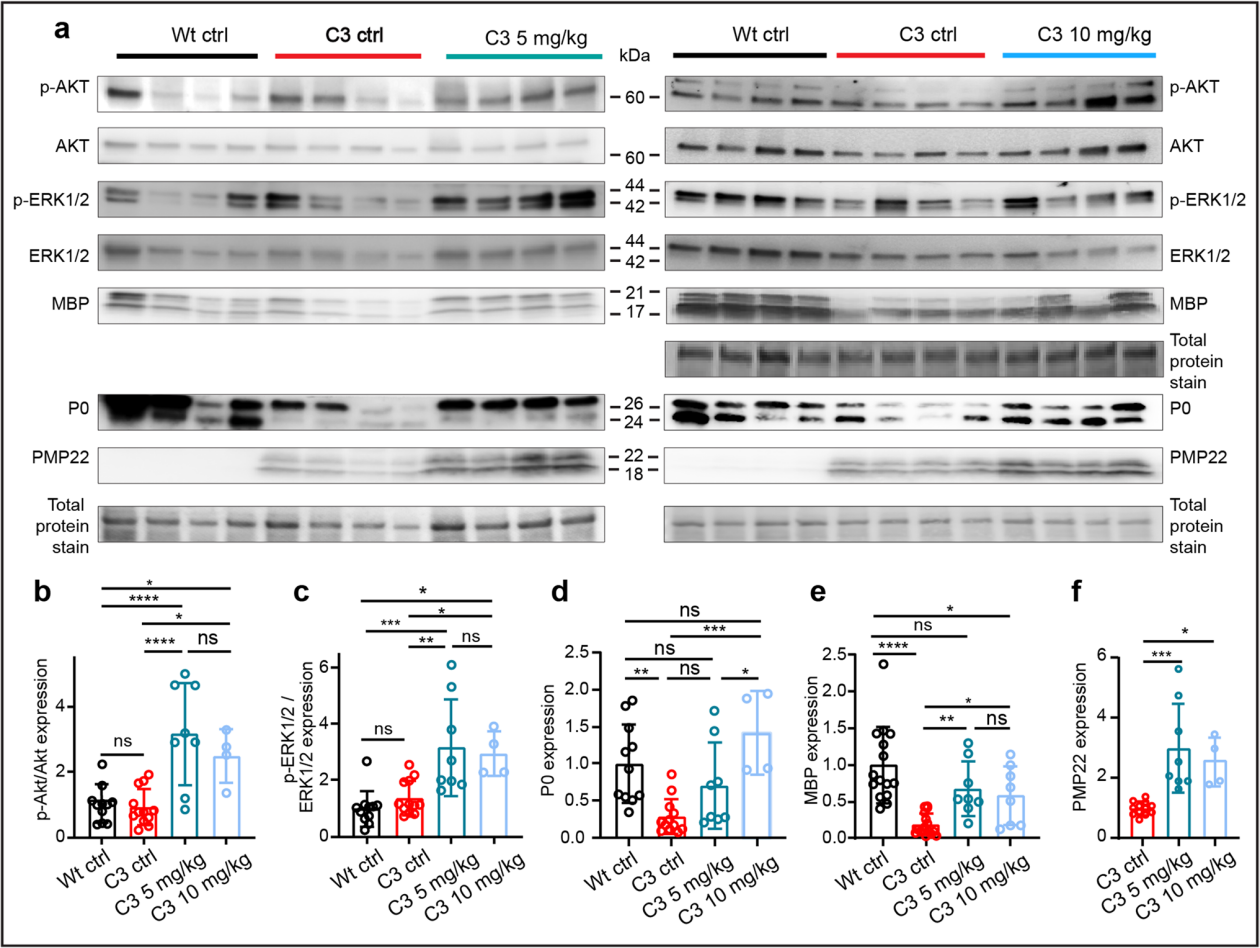
Inhibiting HDAC3 enzyme activity increases AKT-ERK1/2 signaling and myelin proteins during C3 postnatal development. **a** Western blots of p-AKT, AKT, p-ERK, and ERK1/2 in addition to the myelin proteins MBP, P0, and PMP22, in Wt ctrl, C3 ctrl and C3 5 mg/kg (left), and Wt ctrl, C3 ctrl, and 10 mg/kg RGFP966 treated mice (right). **b**–**f** Quantifications of the Western blots. Additional blots used in quantifications relating to C3 5 mg/kg treated mice as well as C3 10 mg/kg treated mice are included in Additional file 2, Fig. S2. All myelin proteins were normalized to total protein stain, which is located below each Western blot. For **b** and **c**, Wt ctrl *n* = 11 mice, C3 ctrl *n* = 12 mice, C3 5 mg/kg *n* = 8 mice, C3 10 mg/kg *n* = 4 mice. For **d**, Wt ctrl *n* = 11 mice, C3 ctrl *n* = 12 mice, C3 5 mg/kg *n* = 8 mice, C3 10 mg/kg *n* = 4 mice. For **e**, Wt ctrl *n* = 15 mice, C3 ctrl *n* = 16 mice, C3 5 mg/kg *n* = 8 mice, C3 10 mg/kg *n* = 8 mice. For **f**, C3 ctrl *n* = 12 mice, C3 5 mg/kg *n* = 8 mice, C3 10 mg/kg *n* = 4 mice. Statistical significance was evaluated in **b**–**e** with a two-way ANOVA with a Tukey’s multiple comparison’s test (*p < 0.05, **p < 0.01, ***p < 0.001, ****p < 0.0001). Statistical significance was evaluated in **f** with one-way ANOVA and Tukey’s multiple comparison’s test (*p < 0.05, **p < 0.01, ***p < 0.001, ****p < 0.0001). In **b**–**d**, data are expressed as fold change from the mean of the Wt ctrl group ± SD. In **f**, data are expressed as fold change from the mean of the C3 ctrl group ± SD, as normalization to PMP22 expression in the Wt ctrl was not possible

As early Schwann cell differentiation capacity appears altered in C3 postnatal day 6 pups (Fig. 1c, d), we treated C3 pups with RGFP966 for 3 weeks following the same treatment scheme as shown in Fig. 2a. Using Western blot analysis of whole sciatic nerve lysates, we observed that C3 mice treated with 5 and 10 mg/kg RGFP966 had significantly upregulated p-AKT, p-ERK1/2, MBP, and PMP22 levels compared to the C3 ctrl mice (Fig. 3a–f; Additional File 2, Figure S2). In C3 10 mg/kg RGFP966 treated mice, P0 was also upregulated in comparison to the C3 ctrl mice (Fig. 3a, d). In the untreated C3 mice versus the Wt mice, we did not observe a major downregulation of p-AKT and p-ERK1/2 expression at this stage of development (Fig. 3a–c). Altogether, our data show that HDAC3i increases factors that are crucial for activation of myelin protein expression.

### HDAC3 Inhibition Improves Electrophysiological Recordings in C3 Mice

**Fig. 4:**
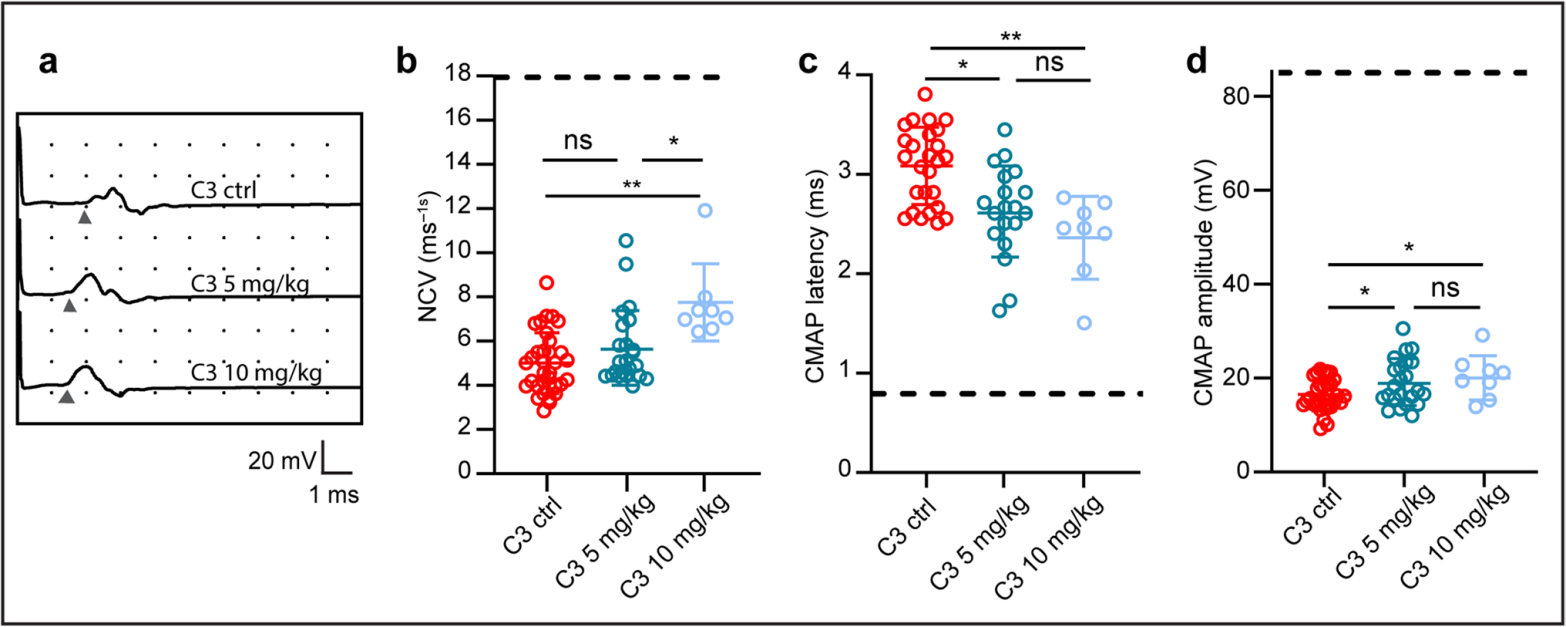
HDAC3 inhibition improves NCVs and CMAP latencies and amplitude recordings in C3 mice. **a** Representative tracings of electrophysiological recordings for the C3 ctrl and for the C3 mice treated with 5 mg/kg and 10 mg/kg of RGFP966. Arrowheads indicate the point at which the potential starts. All electrophysiological tests were conducted during phenotyping between postnatal days 35 and 36. **b** Quantification of the nerve conduction velocity (NCV) recordings from the sciatic nerve. **c** Quantification of CMAP latencies. **d** Quantifications of the CMAP amplitudes. In **b**–**c**, Wt ctrl values are indicated by a black dotted line in each graph. Statistical significance was determined in panel **b** with a Kruskal–Wallis test with Dunn’s multiple comparison’s test, in panels **c** and **d** with a one-way ANOVA with a Tukey’s multiple comparison’s test (*p < 0.05 and **p < 0.01). Mice used in **b**–**d** per group: C3 ctrl = 33, C3 5 mg/kg = 23, and C3 10 mg/kg = 8. Data are expressed in **b**–**d** as mean ± SD

Electrophysiological recordings in the sciatic nerve of C3 mice treated with either 5 or 10 mg/kg HDAC3i showed a dose-dependent improvement in NCVs and in CMAP latencies after HDAC3i treatment (Fig. 4a–c). An improvement in NCVs and CMAP latencies indicate that the speed at which the action potentials propagate has increased, implying that either myelination has increased or alternatively that the Nodes of Ranvier distance has shortened in the HDAC3i treated C3 mice. Assessment of CMAP amplitudes revealed an improvement in both 5 and 10 mg/kg HDAC3i treated C3 groups in comparison to the C3 ctrl group (Fig. 4d). The CMAP amplitude is the summation of a group of almost simultaneous motor nerve action potentials evoked from several muscle fibers in the same surrounding area. Interestingly, Wt pups treated with the same HDAC3i concentrations did not show any significant change in CMAP amplitude or latencies when compared to Wt ctrl mice (see Additional File 3, Figure S3). Collectively, these results show that HDAC3 inhibition increases myelination and axonal action potential propagation in postnatally HDAC3i treated C3 mice.

### HDAC3 Inhibition Upregulates Myelination and Axon Caliber Size in C3 Mice

**Fig. 5:**
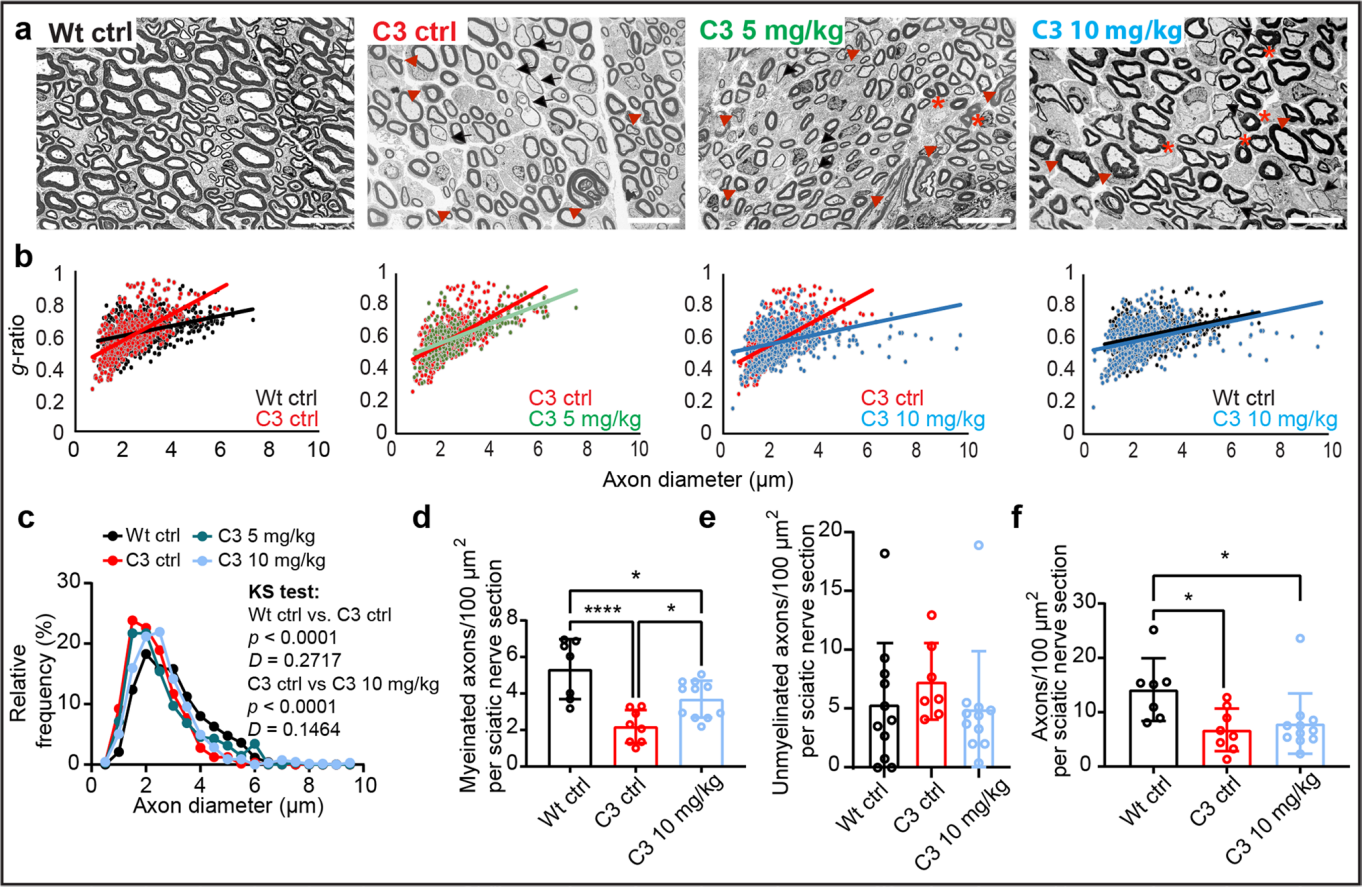
HDAC3 inhibition increases myelination and axon caliber size in C3 mice. **a** Electron microscopy images of the sciatic nerve sections from the Wt ctrl, C3 ctrl, C3 5 mg/kg, and C3 10 mg/kg groups. Scale bar: 10 μm. Schwann cell dysmyelination is indicated by red arrowheads, hypermyelination by red asterisks and hypomyelination by black arrows. Images were taken at the central region of the sciatic nerve. **b** Analysis of *g*-ratios. C3 ctrl mice (red, *n* = 4 mice) compared with Wt ctrl mice (black, *n* = 4 mice). C3 5 mg/kg HDAC3i treated mice (green, *n* = 2) compared to C3 ctrl (second panel) and C3 10 mg/kg RGFP966 treated mice (blue, *n* = 4) compared to the C3 ctrl mice (third panel in from the left). Wt ctrl mice compared to C3 10 mg/kg HDAC3i treated mice (fourth panel to the right). A range of 150–200 fibers per animal were measured at 800 × magnification. **c** Histogram of the relative frequency distribution of the axon diameters. Kolmogorov–Smirnov, two-tailed statistical analysis was used to analyze the difference between the cumulative distribution curves of each sample. Wt ctrl vs C3 ctrl and C3 ctrl vs C3 10 mg/kg were reported in the figure. For C3 ctrl vs C3 5 mg/kg, *p* = 0.0031 and *D* = 0.1191. For Wt ctrl vs C3 10 mg/kg, *p* = < 0.0001 and *D* = 0.1746. For Wt ctrl vs C3 5 mg/kg, *p* = < 0.0001 and *D* = 0.2124. For C3 5 mg/kg vs C3 10 mg/kg, *p* = 0.0119, and *D* = 0.1056. **d** Quantifications of myelinated axons, **e** unmyelinated axons, and **f** total axons per 100 μm^2^ per sciatic nerve section. Number of sciatic nerve sections analyzed **d**–**f** were as follows: Wt ctrl *n* = 7, C3 ctrl *n* = 8, and C3 10 mg/kg *n* = 11. Statistical significance was evaluated in **d** with a one-way ANOVA, Dunn’s multiple comparison’s test (*p < 0.05, ****p < 0.0001). Statistical significance was evaluated in **e** and **f** with a Kruskal–Wallis test, with Dunn’s multiple comparison test (*p < 0.05) to compare groups. Total mice used in **b**–**f** per group: Wt ctrl = 4, C3 ctrl = 4, C3 5 mg/kg = 2, and C3 10 mg/kg = 4. Total number of nerve fibers analyzed in **b** and **c** per group: Wt ctrl = 552, C3 ctrl = 652, C3 5 mg/kg = 350, C3 10 mg/kg = 698

To examine the effect of HDAC3i on myelination and axon caliber size, we examined sciatic nerve biopsies at an electron microscopic level (Fig. 5a). To analyze the myelin–axon relationship, we investigated the *g*-ratio in treated C3 mice, which is the ratio of the inner versus outer layer diameter of the myelin sheath. The larger the outer diameter is with respect to the inner diameter, hence resulting in a lower *g*-ratio, the larger the degree of myelination of that axon. Analysis of *g*-ratios showed a steepening in the regression line in untreated C3 ctrl mice compared to Wt mice, indicating less large myelinated fibers (Fig. 5b). A treatment of 5 mg/kg HDAC3i had a subtle effect on perturbed *g*-ratio distribution, while 10 mg/kg HDAC3i showed a strong improvement on perturbed *g*-ratio distribution, as can be seen when compared to the C3 and Wt ctrl groups (Fig. 5b). Additionally, the relative frequency of axon diameters showed an increase in the frequency of larger axon caliber sizes in 10 mg/kg HDAC3i treated C3 mice compared to untreated C3 mice (Fig. 5c). The frequency of myelinated axons per sciatic nerve section was increased in 10 mg/kg HDAC3i treated C3 mice compared to C3 ctrl mice (Fig. 5d), but no significant changes were detected in the amount of unmyelinated axons or the total amount of axons present (Fig. 5e, f). Myelin thickness was improved in the C3 10 mg/kg treated group compared to the Wt ctrl versus the C3 ctrl group (see Additional File 4, Figure S4). Taken together, these data demonstrate that HDAC3i stimulates myelination and increases the frequency of larger caliber axons in the C3 mice.

### HDAC3 Inhibition Induces a Dose-Dependent Decline in the Motor Performance

**Fig. 6:**
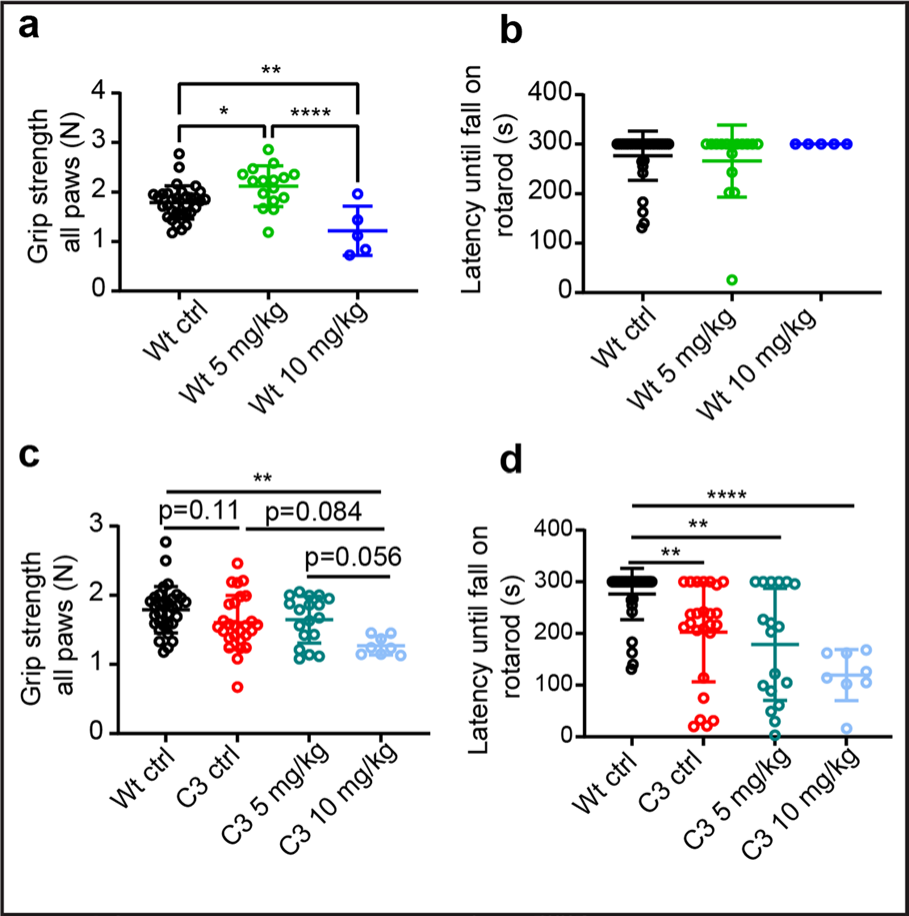
High-dose HDAC3 inhibition causes a decline in the motor performance in Wt and C3 mice. **a** Effect of RGFP966 on overall grip strength was assessed on the Wt mice at postnatal day 30. **b** At the same time-point, the effect of RGFP966 on the motor performance was assessed using rotarod analysis in the Wt mice. For the C3 mouse model (with the Wt ctrl group included as reference), overal grip strength **c**, and rotarod performance **d**, was assessed. Statistical significance was evaluated in **b** and **d** with a Kruskal–Wallis test with Dunn’s multiple comparison’s test (**p < 0.01, ****p < 0.0001). Statistical significance was evaluated in **a** and **c** with a one-way ANOVA with a Tukey’s multiple comparison’s test (*p < 0.05, **p < 0.01, ****p < 0.0001). Mice used in the different groups in panels **a**-**d **per group: Wt ctrl, *n* = 32; Wt 5 mg/kg, *n* = 16; Wt 10 mg/kg, *n* = 5; C3 ctrl, *n* = 33; C3 5 mg/kg, *n* = 23; and C3 10 mg/kg, *n* = 8

We also evaluated the effect of the HDAC3i treatment on the motor phenotype of Wt and C3 mice. At postnatal day 30, the mice were assessed using rotarod and overall grip strength tests. Interestingly, the 5 mg/kg HDAC3i dose treated Wt mice had an increased overall grip strength when compared to Wt ctrl mice, while there was a decrease at the 10 mg/kg concentration (Fig. 6a). Assessment by rotarod showed no obvious alterations in the motor performance of the treated Wt mice versus the untreated Wt mice (Fig. 6b).

When assessing the treated C3 mice, we noticed a similar decline in the overall grip strength when treated with 10 mg/kg dose (Fig. 6c). Moreover, the 10 mg/kg HDAC3i treated C3 mice showed a significant decline in their rotarod performance (Fig. 6d). Taken together, these data indicate that HDAC3i has a dichotomous dose-dependent effect on muscle strength and endurance in Wt and in C3 mice.

In addition, we examined the muscle fiber diameters from the gastrocnemius muscles from Wt and C3 treated and ctrl mice. We observed no alteration in the 10 mg/kg HDAC3i treated Wt mice versus untreated Wt mice (see Additional File 5, Figure S6a, b). Only a subtle reduction in fiber diameters < 20 μm was seen in 10 mg/kg HDAC3i treated C3 mice when compared to the C3 ctrl mice (see Additional File 5, Figure S6b). However, it is unlikely that these differences can account for the observed changes in motor performance.

### HDAC3 Inhibition Increases the Presence of Endoneural Macrophages in C3 Mice

**Fig. 7:**
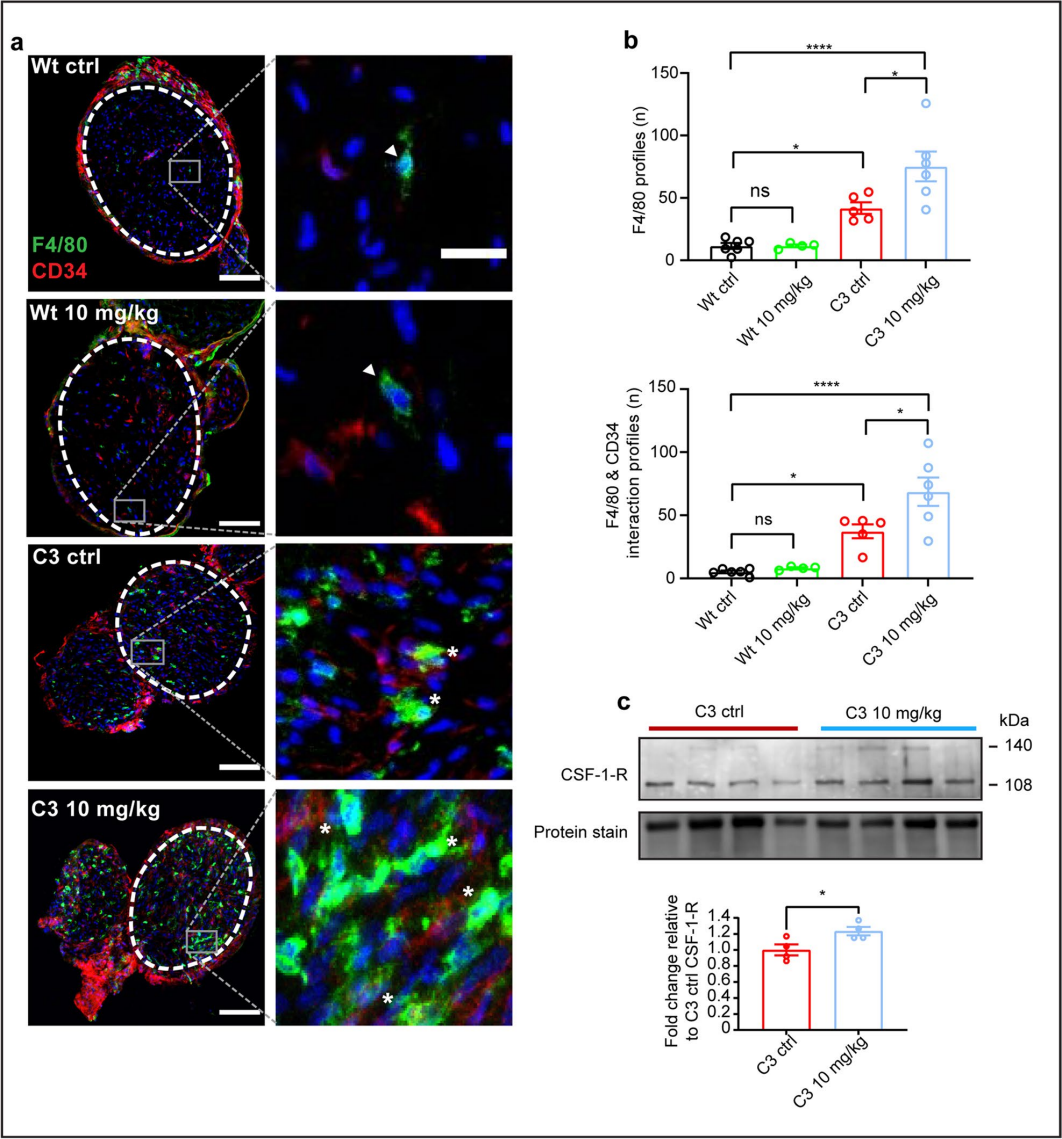
HDAC3 inhibition increases the presence of endoneural macrophages in C3 but not in wild-type mice. **a** Immunohistochemical staining in the brachial plexus nerves of CD34+ cells (fibroblasts) in red, F4/80+ cells (endoneural macrophage) in green, and the nucleus of cells are stained with DAPI in blue. Scale bars: 100 μm. A white dashed oval encompasses the region of the sections analyzed. To the right of each image is a 400% zoomed focus of areas to highlight F4/80+ cells (white arrowheads) and the interaction between CD43+ and F4/80+ cells (white asterisk). Scale bar: 20 µm. **b** Macrophage presence was quantified by the number of F4/80+ cells per slice, and macrophage activation was quantified by the overlap between CD34+ and F4/80+ cells per slice. **c** Western blot analysis of sciatic nerve CSF-1-R protein expression in C3 ctrl and 10 mg/kg treated mice, with quantification of the Western blot below. Western blot quantification is expressed as fold change from C3 ctrl. For **b**, data are represented as the mean of 3–4 slices for each mouse ± SEM. The number of mice analyzed was as follows: Wt ctrl *n* = 6, Wt 10 mg/kg *n* = 4, C3 ctrl *n* = 5, and C3 10 mg/kg *n* = 6. For statistical significance in **b**, a two-way ANOVA with a Tukey’s multiple comparison’s test (*p < 0.05, ****p < 0.0001) was used to compare groups. In **c**, a student’s t-test was used to determine statistical significance (*p < 0.05)

HDAC3 is also known to govern the inflammatory response in macrophages [30, 31]. For demyelinating forms of CMT, such as CMT1A and CMT type 1X (CMT1X), increased presence and activation of endoneural macrophages have been shown to play a role in the pathogenesis [32, 33]. This is due to the demyelination process ongoing in the peripheral nerves, causing excessive myelin debris [34]. The activation of macrophages has been shown—in part—to be orchestrated through the direct interaction of endoneural fibroblasts and macrophages. Similarly, the expression of colony-stimulating factor 1 (CSF-1) by endoneural fibroblasts and its cognate receptor (CSF-1-R) are mediators of the neuroimmune response [32]. Therefore, we examined the presence and activation of macrophages in the brachial plexus nerves of control and 10 mg/kg HDAC3i treated Wt and C3 mice (Fig. 7a–c). Using immunohistochemistry, we identified endoneural fibroblasts as CD34+ cells and F4/80+ cells as a measure of immune cell—macrophage—presence in the nerves of the control and treated mice. The overlap between CD34+ and F4/80+ was used as a measure for the initial steps of macrophage activation. We observed a significant increase in macrophage presence in C3 mice versus Wt mice (Fig. 7a, b), which is in line with what has been observed in the milder C61-PMP22 mouse model of CMT1A [33]. Likewise, the interaction of macrophages and fibroblasts was increased in the C3 versus Wt mice (Fig. 7a, b). In the 10 mg/kg HDAC3i treated C3 mice, there was a further increase in macrophage presence and interaction with endoneural fibroblasts (Fig. 7a, b). The presence of macrophages or the interaction with endoneural fibroblasts was not altered in 10 mg/kg treated Wt mice (Fig. 7a, b). As the presence and interaction of macrophages with endoneural fibroblasts was increased in C3 10 mg/kg HDAC3i treated mice, we checked the expression levels of CSF-1-R protein in sciatic nerve lysates, as a further indicator of macrophage activation. Here, the 10 mg/kg HDAC3i treated C3 mice showed a subtle, but significant increase in CSF-1-R protein expression in the sciatic nerve compared to untreated C3 mice (Fig. 7c). Overall, these data indicate that the high-dose HDAC3i treatment stimulates the presence of macrophages in the peripheral nerves of C3 mice, which may be beneficial due to the improvement in histological observations and electrophysiological recordings or has a minor adverse effect in the nerve which is overcompensated by the positive effect of HDAC3i in the nerve.

## Discussion

Duplication of the *PMP22* gene in CMT1A dysregulates myelination of the peripheral nerves during early postnatal development. Later in life, this induces slowly progressing secondary peripheral axonal loss and muscle atrophy, which manifests in muscle weakness and sensory impairment. Stabilization of patient phenotypes occurs with only incremental changes throughout lengthy periods, unless environmental, lifestyle, medication, or similar factors have a disease-altering effect. Using the C3 mouse model, we observed postnatal Schwann cell defects and a progressive neuropathy that partially stabilized later during life. During the active stage of myelination, inhibiting the HDAC3 enzyme’s catalytic activity, one of the key regulators of myelin homeostasis, activated ERK1/2 and AKT signaling and upregulated MBP, P0, and PMP22 protein synthesis in these mice. On the histological level, this therapeutic approach significantly increased myelination and axonal diameters. Moreover, the stimulation of these pathways improved several electrophysiological parameters in the nerves. However, a dichotomous dose-dependent effect on the in vivo motor phenotype was observed between the Wt and C3 treated mice. In addition, a further increase of macrophage presence in the peripheral nerves was specifically observed in C3 10 mg/kg treated mice.

The postnatal deficits in Schwann cell development observed in the C3 mice are similar to the ones described in the CMT1A rat model [8]. CMAP amplitude deficits were most severe early in development but improved partially with time until a plateau in improvement was reached, in line with what has been published in the original study [27]. However, we discovered that C3 mice harbor 5 copies of human *PMP22*, which is different from the previously reported 3–4 copies of human *PMP22* [27]. This could be due to differences in the improved detection sensitivity of the current method, the ddPCR, versus quantitative PCR used in the original study [27].

As a reduction in myelination and axonal diameter are core features in the pathology of CMT1A disease [25], therapeutic strategies aimed at mitigating the disease phenotype typically revolve around stimulating myelination [7, 8, 35] or downregulating the levels of PMP22 [36].

In this study, we investigated whether HDAC3 inhibition could be a valuable therapeutic approach for CMT1A by stimulating myelination in the C3 mouse model. He et al. showed that downregulating HDAC3 activity through pharmacological inhibition or genetic ablation promotes remyelination after nerve injury [18]. Moreover, these authors extensively showed that downregulating HDAC3 led to an upregulation of the myelin transcriptional network, myelin protein expression, and stimulation of PI3K–AKT and MAPK/ERK1/2 signaling pathways [18]. Another study by Rosenberg et al. demonstrated that HDAC3 was a key enzyme regulating myelin homeostasis [29]. These authors showed that *Hdac3*^−/−^ Schwann cells initiated myelination normally in early postnatal development but overshoot myelination later in postnatal development. Furthermore, Rosenberg et al. showed that mouse *Hdac3*^−/−^ Schwann cells remained in a myelin biogenic state [29]. In both studies, *g*-ratios were significantly lowered which represent a higher degree of myelin ratio with regard to their associated axons [18, 29].

To validate that HDAC3 inhibition stimulates myelination in vivo, we tested several markers for myelination in Wt and C3 mice that were treated with RGFP966 for 3 weeks, starting at postnatal day 6. The PI3K–AKT pathway is the key signaling pathway regulating the proliferation, survival, and differentiation of early developmental Schwann cells at the initial stages of myelination [11]. Fledrich et al. demonstrated that PI3K–AKT signaling is continuously downregulated in the sciatic nerves of the CMT1A rat model at postnatal day 1 to 84 [8]. Accompanying the dysregulated myelination, this PI3K–AKT signaling reduction indicates that CMT1A Schwann cells have differentiation difficulties at the initial steps of myelination. In the same study, the authors showed that CMT1A rat Schwann cells had an initial reduction in the MAPK/ERK1/2 signaling pathways (< postnatal day 6) followed by a sustained increase in signaling later in postnatal development (> postnatal day 18) [8].

In this study, we showed that 6-month-old C3 mice have a significant downregulation of AKT-ERK1/2 signaling pathways and the expression of MBP and P0 in comparison to Wt littermate controls. Additionally, we showed that reducing HDAC3 catalytic enzymatic activity in Wt mice stimulated downstream AKT and ERK1/2 signaling pathways and upregulated MBP expression in the sciatic nerves. In C3 mice treated with the HDAC3i, we detected a similar increase in the expression of the myelin proteins MBP, P0, and PMP22. Furthermore, although the major myelin proteins were upregulated in both HDAC3i treatment conditions, PMP22 protein levels did not stabilize and were further increased in the treatment conditions. This may limit HDAC3i’s therapeutic potential in patients carrying extra copies of myelin associated genes, as myelination as a whole seems to be stimulated and not stabilized.

At the stage in development in which the C3 mice were assessed after treatment, we did not observe significant reduction in AKT or ERK1/2 signaling. However, the HDAC3i treatment in the C3 mice significantly increased AKT signaling in comparison to C3 ctrl mice and ERK1/2 signaling in comparison to C3 and even to the Wt ctrl mice. Sustained ERK1/2 signaling has been shown to enhance myelin growth in Schwann cells and oligodendrocytes [37], and this could explain the significantly improved myelination in the C3 mice treated with the HDAC3i. Similarly, this could be the reason for the improvement of the electrophysiological recordings, *g*-ratios, myelin thickness, and axon caliber size. However, excessive ERK1/2 signaling has been shown to be detrimental to myelination [38–40], which could worsen the CMT-phenotype if maintained over time.

Interestingly, the dichotomous dose-dependent effect observed on the motor phenotype in Wt mice was most likely not due to neuromuscular junction connection or muscle fiber size but might be due to a change in skeletal muscle metabolism. Mice with skeletal muscle-specific knockout of *Hdac3* (HDAC3-SkMKO mice) have been shown to have severe systemic insulin resistance [41]. Indeed, despite reducing muscle force, depletion of *Hdac3* from the skeletal muscle of these mice resulted in enhanced endurance and resistance to muscle fatigue motor phenotype [41]. However, these authors do highlight that the endurance phenotype was characterized using a low-intensity exercise protocol. As more vigorous exercise is also more demanding on muscle strength, which is compromised in HDAC3-depleted muscles, this could lead to decreased exercise tolerance in HDAC3-SkMKO mice [41]. In a follow-up study using a knock-in mouse model with mutations in the deacetylase-activating domain (DAD) of both nuclear co-repressors NCOR1 and SMRT (NS-DADm), which are required for HDAC3 deacetylase enzyme activity [42], Song et al. demonstrated that HDAC3 enzymatic activity regulates skeletal muscle fuel metabolism [43]. Here, the authors found that skeletal muscle from NS-DADm mice showed lower force generation, enhanced fatigue resistance, and reduced muscle mass during aging, without major changes in the muscle fiber-type composition or mitochondrial protein content compared to control mice [43].

In the current study, we found that Wt mice treated with a low-dose HDAC3i had increased grip strength, but at a high dose, this resulted in a reduction of grip strength, in line with studies of mice with complete depletion of HDAC3 or its enzymatic activity from skeletal muscle [41, 43]. The increase and decrease of grip strength observed in the Wt mice treated with low- and high-dose HDAC3i, respectively, illustrate the balancing role HDAC3's enzymatic activity plays in muscle force generation. For the assessment of motor endurance, we utilized a moderate-to-high intensity rotarod assessment (constant speed of 15 rpm for 300 s) in which no observable alteration in endurance performance was noted in Wt HDAC3i treated mice. Interestingly, an opposing dose–response was found in the C3 mice, where a strong decrease in rotarod motor endurance was found in the high-dose HDAC3i treated mice compared to C3 ctrl mice. For overall grip strength, a similar reduction in force generation was found in the high-dose HDAC3i C3 mice as in the Wt mice. These subtle differences in phenotypic response to HDAC3i highlight transgene differences in the C3 mice in comparison to Wt mice.

In demyelinating neuropathies, such as CMT1A and CMT1X, elevated macrophage infiltration and activation in the peripheral nerves have been shown to be critically involved in the pathogenesis [32–34]. Initially, macrophages play an essential role during Wallerian degeneration, where they help remove myelin debris, which in itself is an inhibitor of axonal regeneration, and they mitigate the immune response in a controlled manner [34, 44]. However, during chronic dysregulation of myelination, macrophages have been shown to be disease-amplifying agents [33, 34]. We found a higher number of F4/80+ macrophages in the nerves of C3 ctrl versus Wt ctrl mice, and this was higher in the C3 10 mg/kg treated group versus both the C3 ctrl and Wt ctrl groups. Activation of macrophages has been shown to be partially mediated through contact with endoneural fibroblasts and the expression of CSF-1 protein [32, 45]. Interestingly, HDAC3 is a dual transcriptional activator and repressor of the innate immune system, with a non-canonical deacetylase function that orchestrates the inflammatory response in macrophages [31]. Macrophage-specific knockout of *Hdac3* has been shown to dampen the immune response through alternative activation of macrophages [30, 46]. We found that HDAC3i led to macrophage presence and activation to be exacerbated in treated C3 mice in comparison to untreated C3 mice. However, as the activation of macrophages was partially associated to CSF-1-R increased expression, it should be noted that this may be due to the overall increase in macrophage presence in the nerve. As we did not find an alteration in the presence of macrophages in the brachial plexus nerves of Wt mice treated with a HDAC3i, this indicates that the difference in macrophage presence in the nerve was neuropathic-specific. Additionally, this also rules out the possibility that neuroinflammation was the causative factor in the reduction of the motor phenotype, and that this reduction was most likely a HDAC3 muscle-specific phenotype.

In conclusion, we discovered that pharmacological inhibition of HDAC3 at the active stage of myelination in CMT1A mice can stimulate myelination and improve the electrophysiological recordings but can also stimulate the presence of macrophages in the peripheral nerves. Interestingly, our results demonstrated a dichotomous motor phenotype between Wt and C3 mice in response to HDAC3 inhibition, which strongly indicates that correct dosing is of crucial importance for HDAC3i—or broad-spectrum HDACi—when reaching a clinical setting for neurological or other disorders.

## Supplementary Information

Below is the link to the electronic supplementary material.

Fig. S1 ESM 1(PDF 69.3 kb).
Figure S1. Structure of the selective histone deacetylase 3 (HDAC3) inhibitor RGFP966. The benzamide-based HDAC3 inhibitor RGFP966 has a > 200 fold selectivity towards HDAC3 versus HDAC1 and HDAC2 [3]. Image was created using ChemDraw 16.0 software (PNG 40 kb)

High resolution image (TIF 5672 kb).

Fig. S2 ESM 2 (PNG 285 kb).
Figure S2. Additional Western blots used for quantifications in figure 3. **a)** Western blots of p-AKT, AKT, p-ERK, ERK1/2 in addition to the myelin proteins MBP, P0, and PMP22, in Wt ctrl, C3 ctrl, and C3 5 mg/kg RGFP966 treated mice. * indicates C3 5 mg/kg RGFP966 treated mice on the blot. Total protein stain was used for normalization. **b)** Western blot of MBP and corresponding protein stain in Wt ctrl, C3 ctrl, and C3 10 mg/kg RGFP966 treated mice. **c)** Western blot showing PMP22 at normal exposure and at a higher exposure to demonstrate that signal was reached in Wt ctrl conditions, but unusable for quantifications when compared to C3 mice. Samples are Wt ctrl, C3 ctrl, and C3 10 mg/kg RGFP966 treated mice as seen in figure 3. This figure relates to figure 3 of the main text (PNG 285 kb)

High resolution image (TIF 8191 kb).

Fig. S3 ESM 3 (PNG 46 kb).
Figure S3. HDAC3 inhibition did not significantly increase electrophysiological recordings in Wt mice. **a)** Quantification of the CMAP amplitude and **b)** latencies in Wt mice treated with HDAC3 inhibitor. Statistical significance was determined in **a)** One-way ANOVA and Tukey’s multiple comparison’s test and for b) with a Kruskal-Wallis test with Dunn’s multiple comparison’s test. Mice used in a-b) per group: Wt ctrl = 18, Wt 5 mg/kg = 19, Wt 10 mg/kg = 5. Data are expressed in a-b) as mean ± S.D. This figure relates to figure 4 of the main text (PNG 46 kb)

High resolution image (TIF 4005 kb).

Fig. S4 ESM 4 (PNG 50 kb).
Figure S4. HDAC3 inhibition improves myelin thickness in C3 high dose treated mice. Myelin thickness measurements from electron micrographs from Fig. 5. Total mice used in per group: Wt ctrl = 4, C3 ctrl = 4, C3 5 mg/kg = 2, C3 10 mg/kg = 4. Total number of nerve fibers analyzed per group: Wt ctrl = 552, C3 ctrl = 652, C3 5 mg/kg = 350, C3 10 mg/kg = 698. For statistical significance, a One-way ANOVA with a Tukey’s multiple comparison’s test (* p < 0.05) was used to compare groups. Data are expressed as mean ± S.D. Statistics was not performed on the C3 5 mg/kg group (PNG 50 kb)

High resolution image (TIF 5357 kb).

Fig. S5 ESM 5 (PNG 854 kb).
Figure S5. HDAC3 inhibition has no major effect on muscle fiber diameter size. **a)** Wheat germ agglutinin was used to stain the plasma membrane of the muscle fibers from the gastrocnemius muscle from different mice. Scale bar:100 μm. **b)** Frequency distribution analyses of all measured gastrocnemius muscle fibers per group. For statistical significance, Kolmogorov–Smirnov (KS) test was conducted (* p < 0.05, ** p < 0.01, *** = p < n0.001, and **** = p < 0.0001), with between 3-4 mice per group, with 5 gastrocnemius sections per animal, and between 2095-4186 muscle fibers were analyzed per group. This figure relates to figure 7 of the main text (PNG 854 kb)

High resolution image (TIF 7935 kb).

## Data Availability

All relevant data of the present manuscript are available from the corresponding authors on reasonable request.
