# Supplementary figures and images for: HDAC3 Inhibition Stimulates Myelination in a CMT1A Mouse Model
Source: Mol Neurobiol. 2022 Mar 23;59(6):3414–30. doi: 10.1007/s12035-022-02782-x (PMC9148289; doi:10.1007/s12035-022-02782-x)

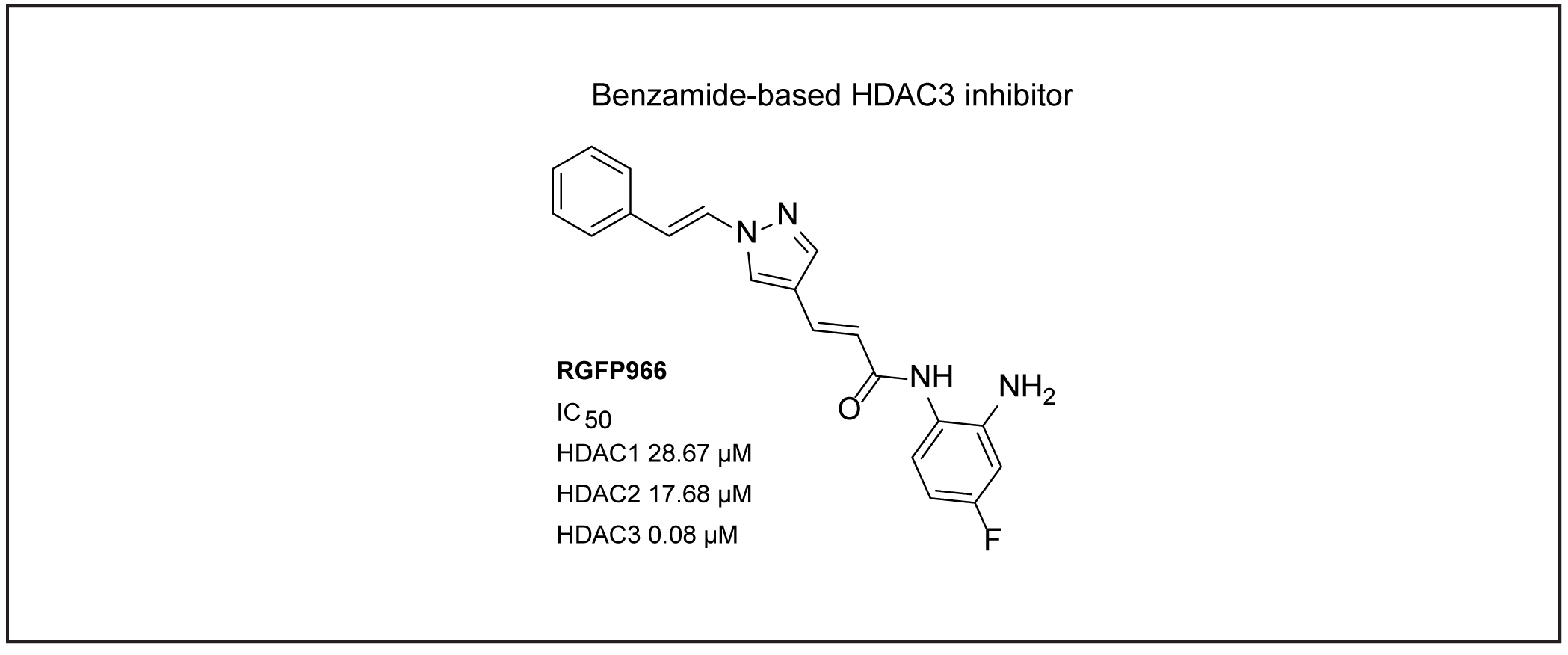

Supplement: Supplementary file 1 — ESM 1(PDF 69.3 kb). Figure S1. Structure of the selective histone deacetylase 3 (HDAC3) inhibitor RGFP966. The benzamide-based HDAC3 inhibitor RGFP966 has a > 200 fold selectivity towards HDAC3 versus HDAC1 and HDAC2 [3]. Image was created using ChemDraw 16.0 software (PNG 40 kb) [file 12035_2022_2782_Fig8_ESM.png]

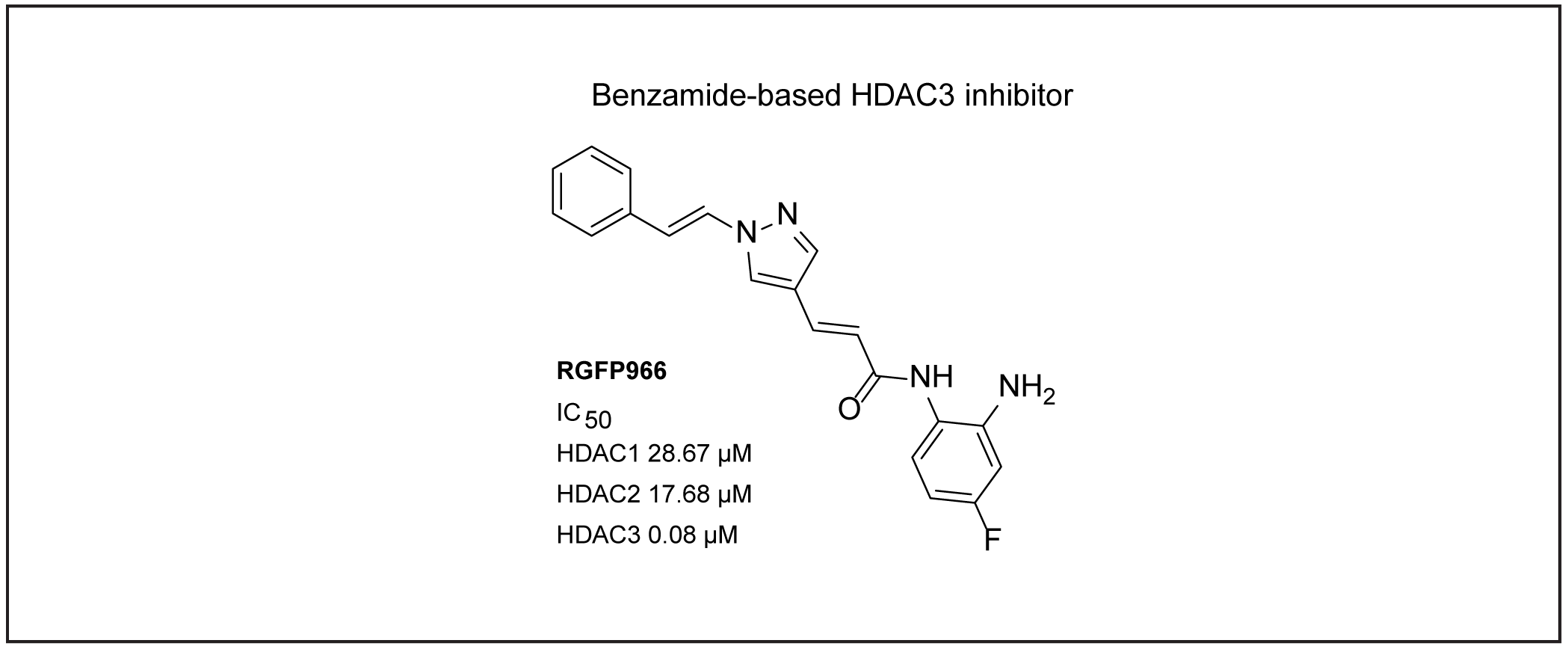

Supplement: Supplementary file 2 — High resolution image (TIF 5672 kb). [file 12035_2022_2782_MOESM1_ESM.tif]

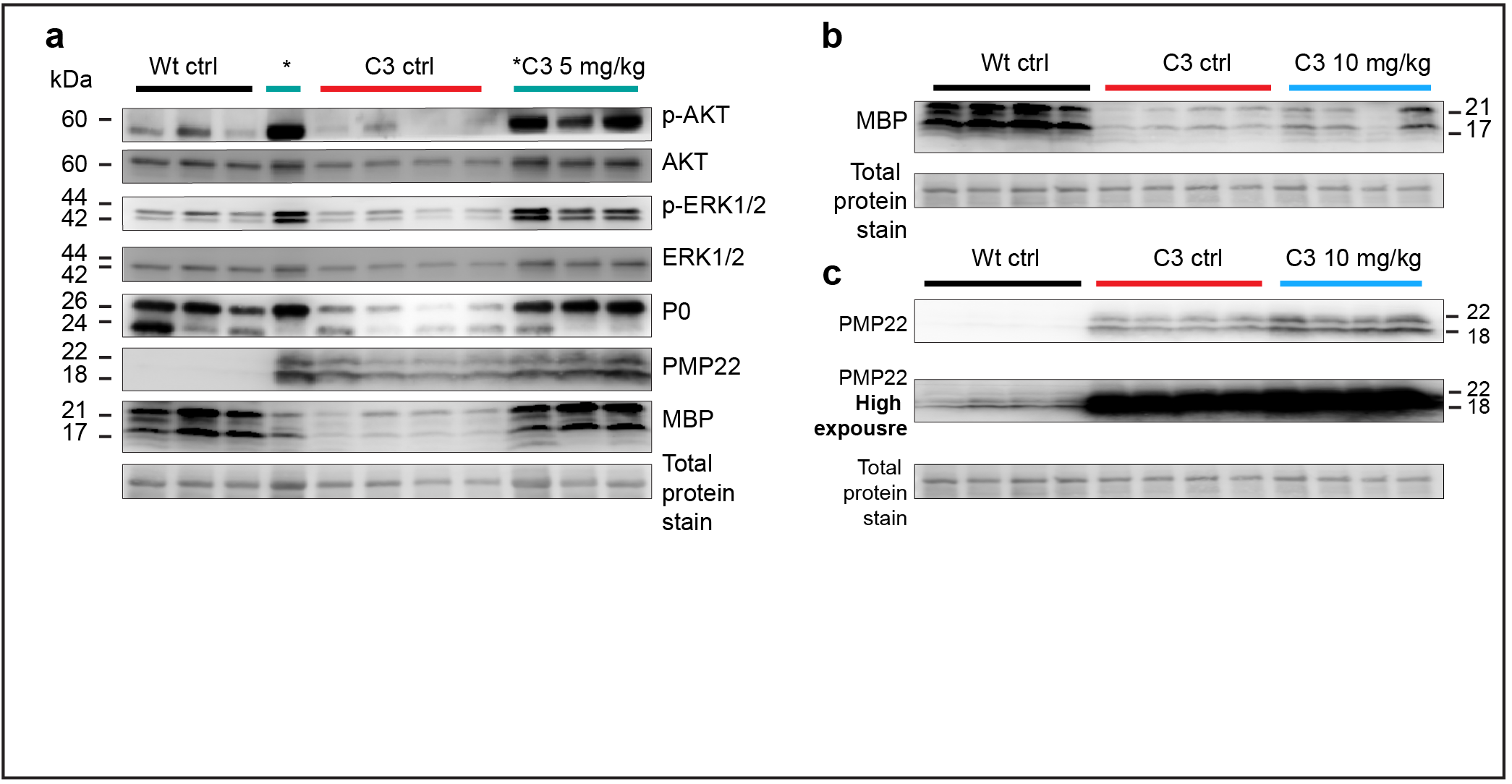

Supplement: Supplementary file 3 — ESM 2 (PNG 285 kb). Figure S2. Additional Western blots used for quantifications in figure 3. a) Western blots of p-AKT, AKT, p-ERK, ERK1/2 in addition to the myelin proteins MBP, P0, and PMP22, in Wt ctrl, C3 ctrl, and C3 5 mg/kg RGFP966 treated mice. * indicates C3 5 mg/kg RGFP966 treated mice on the blot. Total protein stain was used for normalization. b) Western blot of MBP and corresponding protein stain in Wt ctrl, C3 ctrl, and C3 10 mg/kg RGFP966 treated mice. c) Western blot showing PMP22 at normal exposure and at a higher exposure to demonstrate that signal was reached in Wt ctrl conditions, but unusable for quantifications when compared to C3 mice. Samples are Wt ctrl, C3 ctrl, and C3 10 mg/kg RGFP966 treated mice as seen in figure 3. This figure relates to figure 3 of the main text (PNG 285 kb) [file 12035_2022_2782_Fig9_ESM.png]

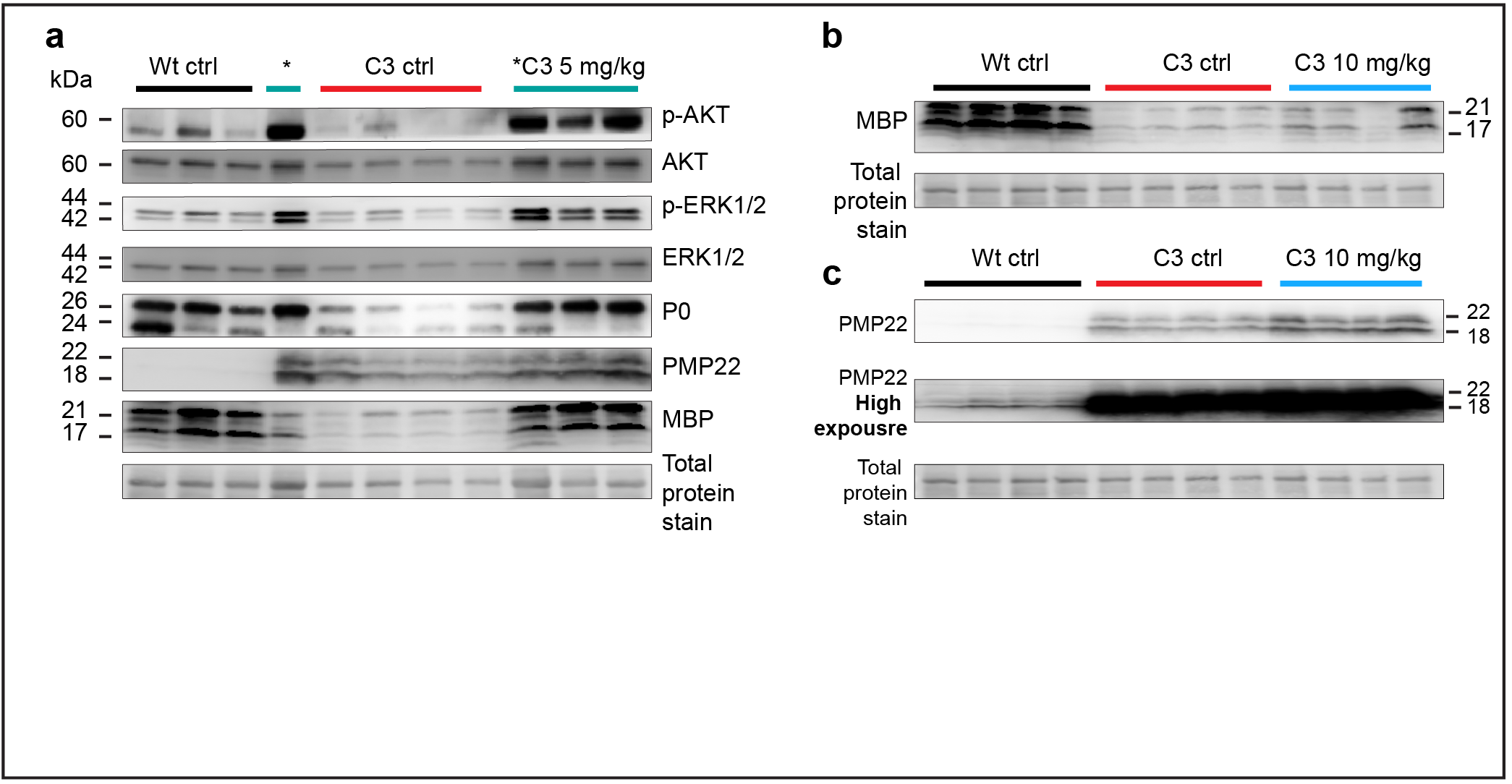

Supplement: Supplementary file 4 — High resolution image (TIF 8191 kb). [file 12035_2022_2782_MOESM2_ESM.tif]

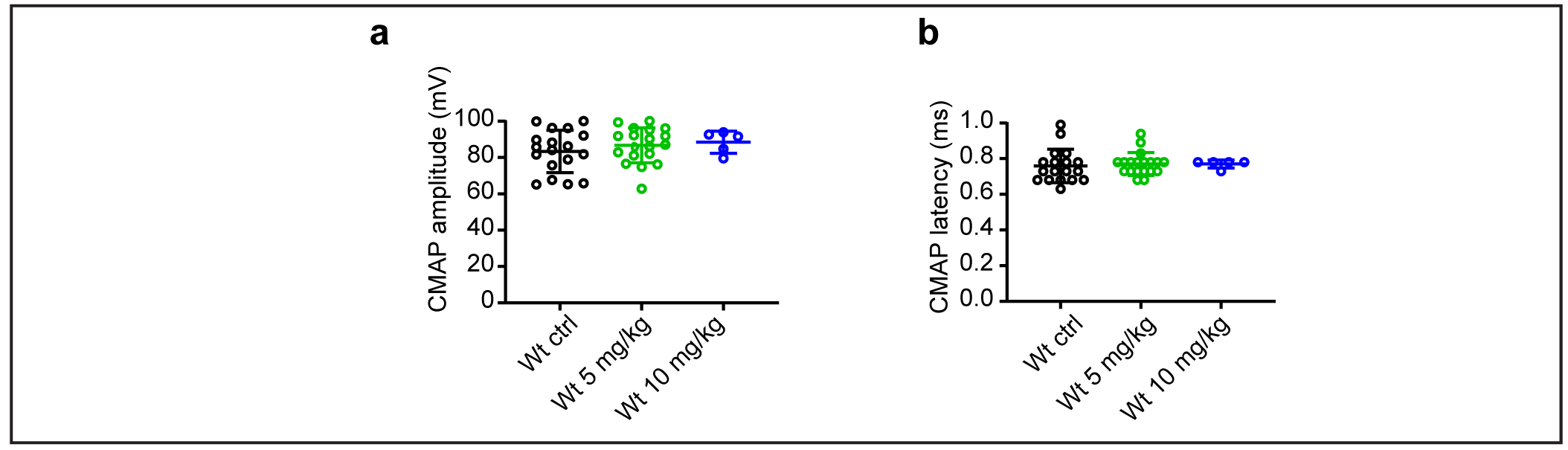

Supplement: Supplementary file 5 — ESM 3 (PNG 46 kb). Figure S3. HDAC3 inhibition did not significantly increase electrophysiological recordings in Wt mice. a) Quantification of the CMAP amplitude and b) latencies in Wt mice treated with HDAC3 inhibitor. Statistical significance was determined in a) One-way ANOVA and Tukey’s multiple comparison’s test and for b) with a Kruskal-Wallis test with Dunn’s multiple comparison’s test. Mice used in a-b) per group: Wt ctrl = 18, Wt 5 mg/kg = 19, Wt 10 mg/kg = 5. Data are expressed in a-b) as mean ± S.D. This figure relates to figure 4 of the main text (PNG 46 kb) [file 12035_2022_2782_Fig10_ESM.png]

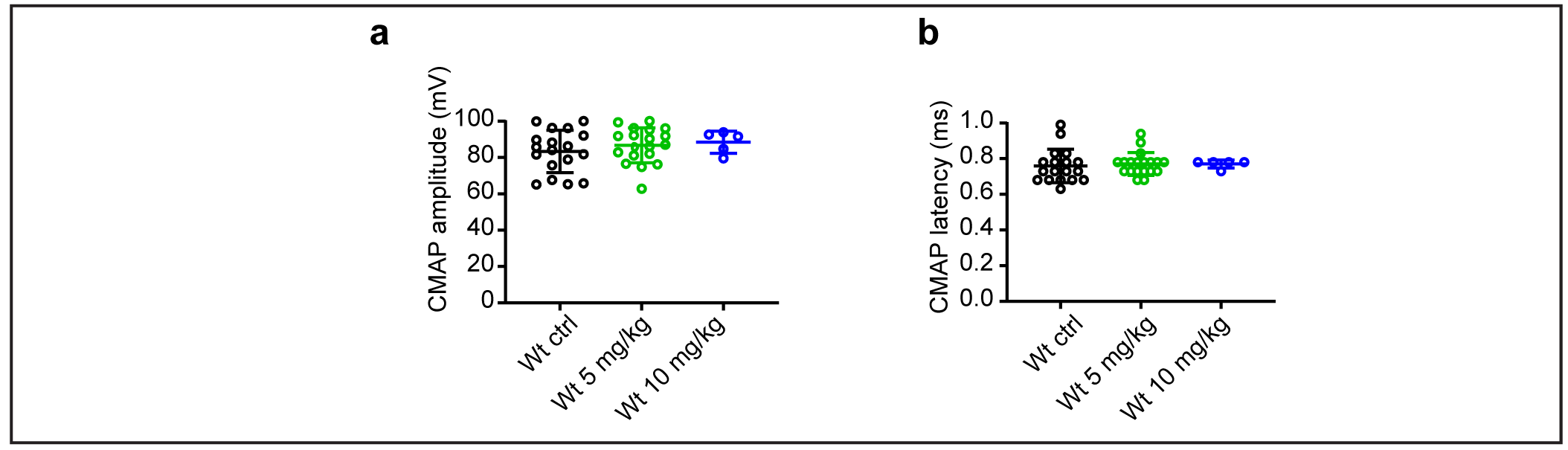

Supplement: Supplementary file 6 — High resolution image (TIF 4005 kb). [file 12035_2022_2782_MOESM3_ESM.tif]

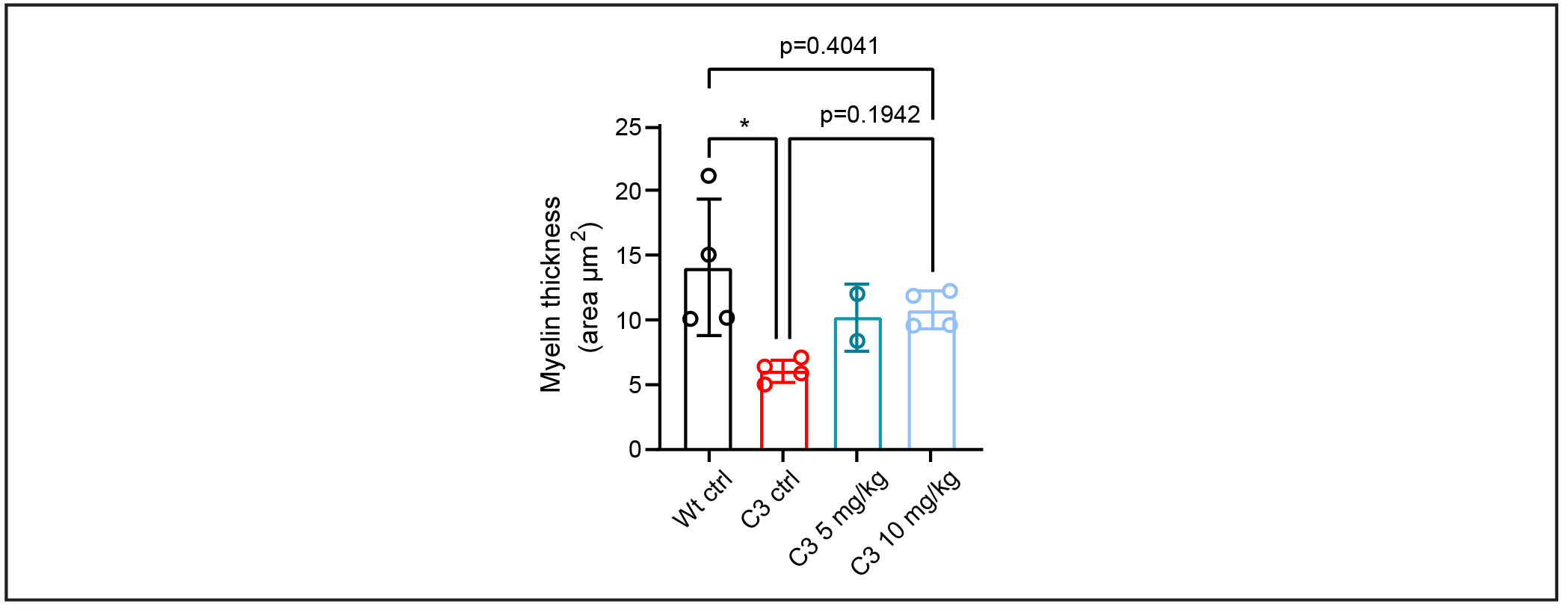

Supplement: Supplementary file 7 — ESM 4 (PNG 50 kb). Figure S4. HDAC3 inhibition improves myelin thickness in C3 high dose treated mice. Myelin thickness measurements from electron micrographs from Fig. 5. Total mice used in per group: Wt ctrl = 4, C3 ctrl = 4, C3 5 mg/kg = 2, C3 10 mg/kg = 4. Total number of nerve fibers analyzed per group: Wt ctrl = 552, C3 ctrl = 652, C3 5 mg/kg = 350, C3 10 mg/kg = 698. For statistical significance, a One-way ANOVA with a Tukey’s multiple comparison’s test (* p < 0.05) was used to compare groups. Data are expressed as mean ± S.D. Statistics was not performed on the C3 5 mg/kg group (PNG 50 kb) [file 12035_2022_2782_Fig11_ESM.png]

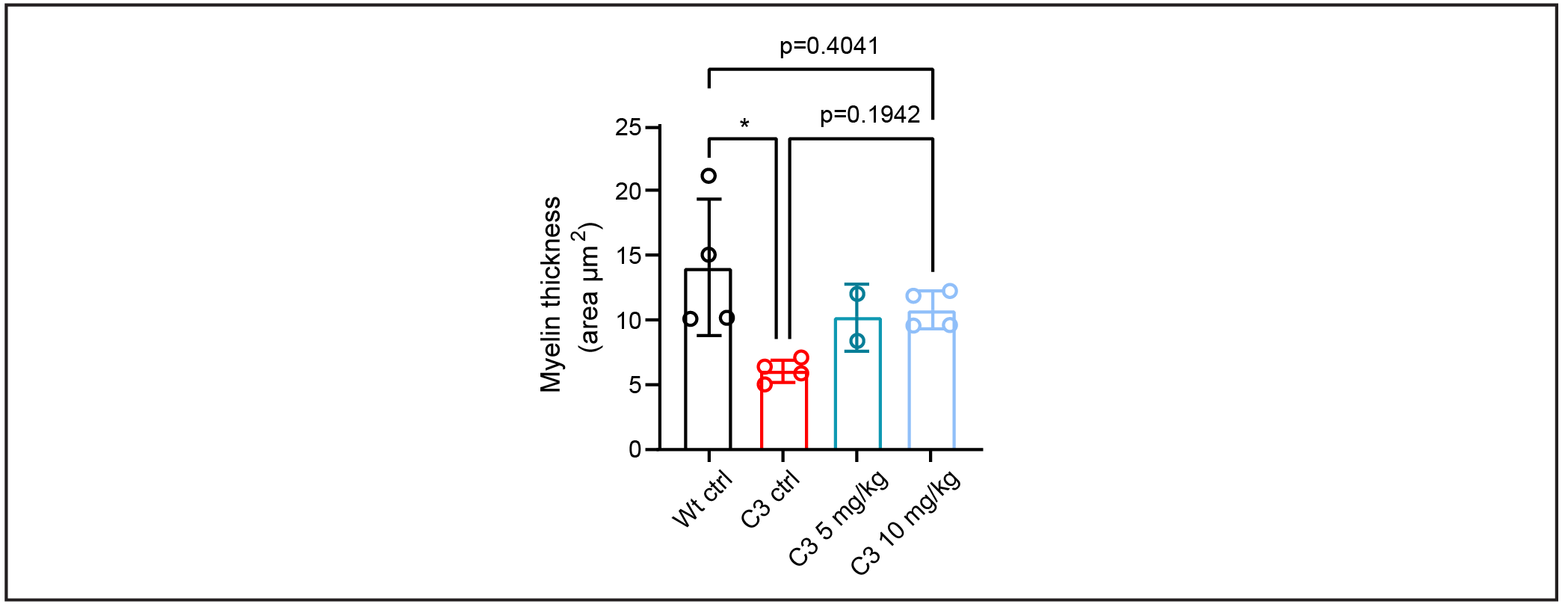

Supplement: Supplementary file 8 — High resolution image (TIF 5357 kb). [file 12035_2022_2782_MOESM4_ESM.tif]

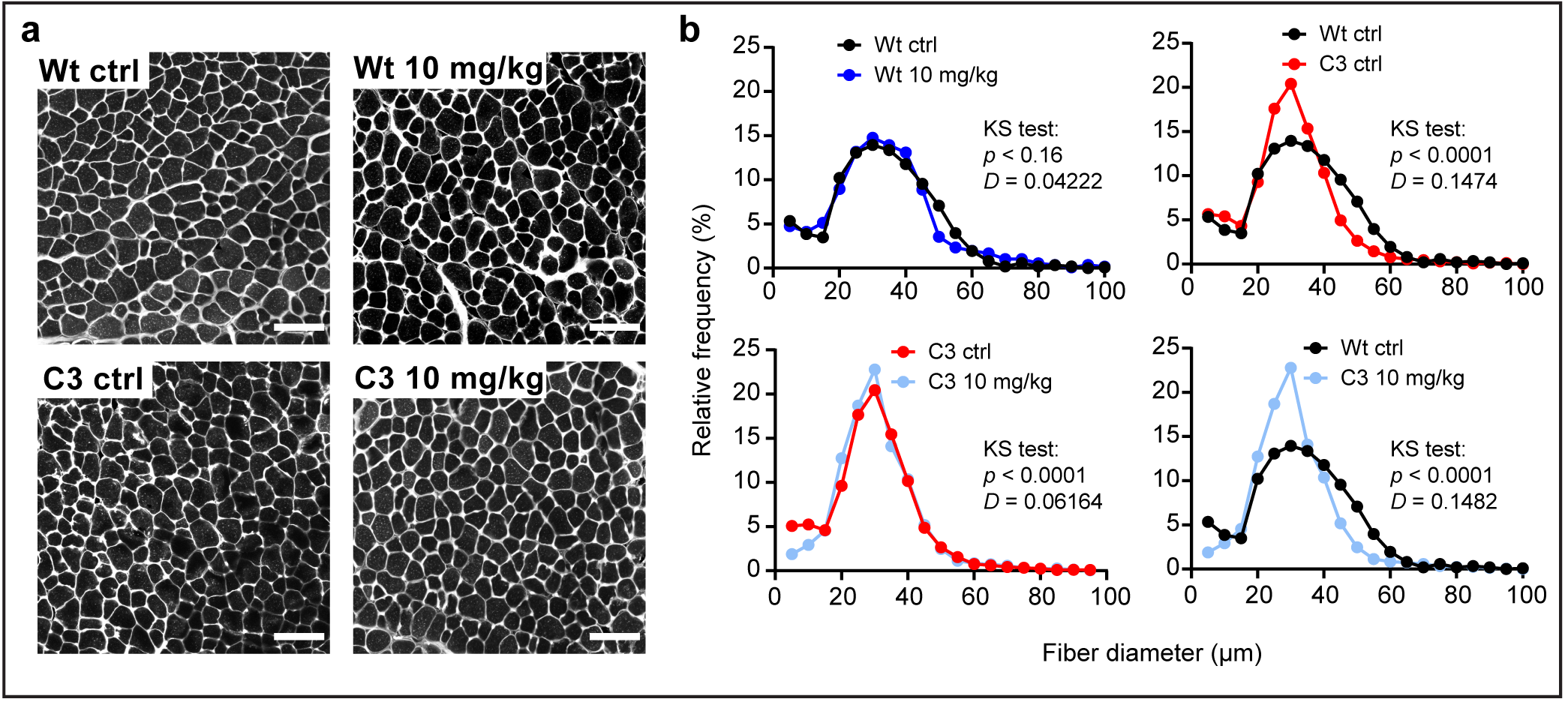

Supplement: Supplementary file 9 — ESM 5 (PNG 854 kb). Figure S5. HDAC3 inhibition has no major effect on muscle fiber diameter size. a) Wheat germ agglutinin was used to stain the plasma membrane of the muscle fibers from the gastrocnemius muscle from different mice. Scale bar:100 μm. b) Frequency distribution analyses of all measured gastrocnemius muscle fibers per group. For statistical significance, Kolmogorov–Smirnov (KS) test was conducted (* p < 0.05, ** p < 0.01, *** = p < n0.001, and **** = p < 0.0001), with between 3-4 mice per group, with 5 gastrocnemius sections per animal, and between 2095-4186 muscle fibers were analyzed per group. This figure relates to figure 7 of the main text (PNG 854 kb) [file 12035_2022_2782_Fig12_ESM.png]

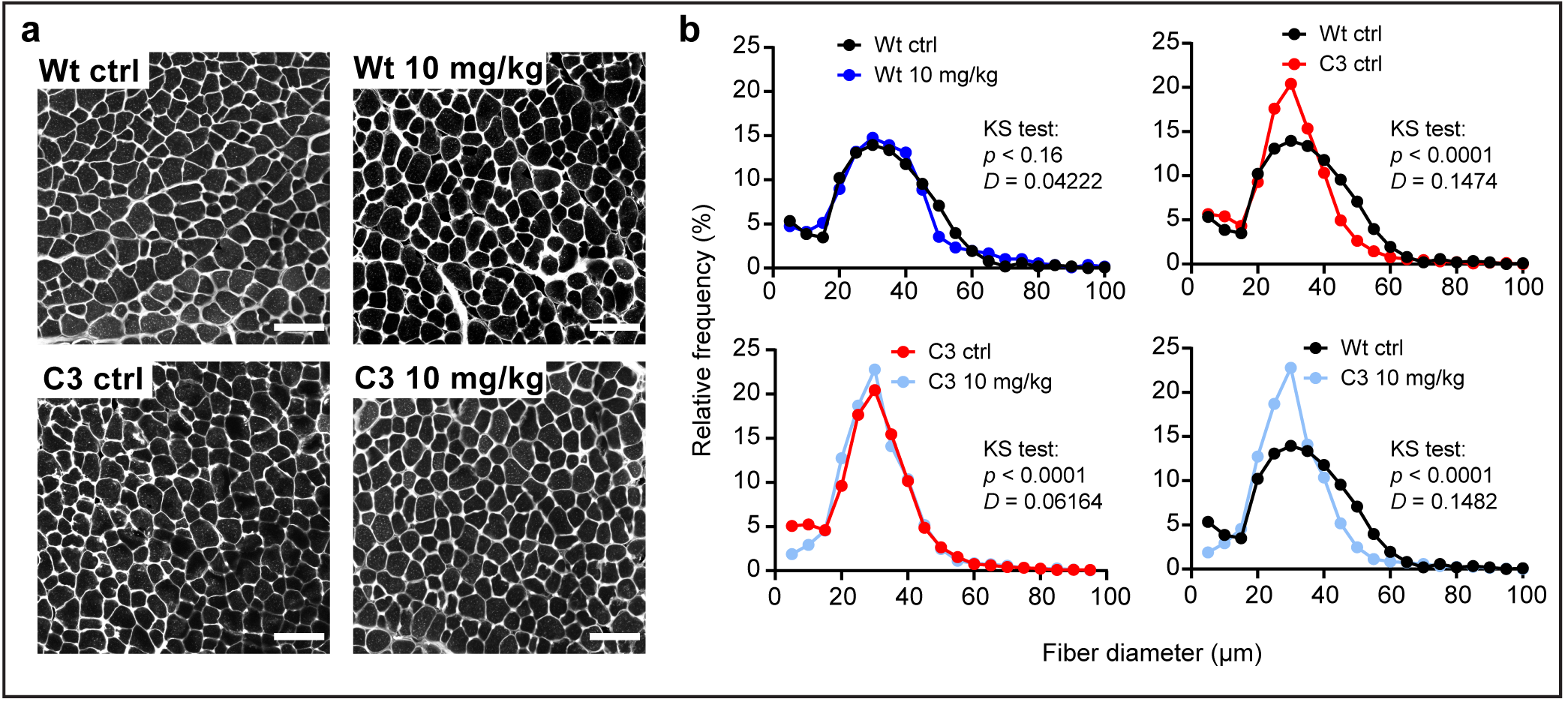

Supplement: Supplementary file 10 — High resolution image (TIF 7935 kb). [file 12035_2022_2782_MOESM5_ESM.tif]
